# Enhanced Prenatal Care Models and Postpartum Depression: The EMBRACE Randomized Clinical Trial

**DOI:** 10.1001/jamanetworkopen.2025.59883

**Published:** 2026-02-16

**Authors:** Jennifer N. Felder, Daisy León-Martínez, Deborah Karasek, Venise Curry, Kristin Carraway, Patience A. Afulani, Bridgette Blebu, Brittany Chambers-Butcher, Kimberly Coleman-Phox, Bethany J. Simard, Cinthia Blat, Mary A. Garza, Charles E. McCulloch, Miriam Kuppermann

**Affiliations:** 1 Department of Psychiatry and Behavioral Sciences, University of California, San Francisco; 2 Osher Center for Integrative Health, University of California, San Francisco; 3 Department of Obstetrics, Gynecology and Reproductive Sciences, University of California, San Francisco; 4 School of Public Health, Oregon Health & Science University, Portland; 5 Central Valley Health Policy Institute, California State University, Fresno; 6 Department of Epidemiology and Biostatistics, University of California, San Francisco; 7 The Lundquist Institute for Biomedical Innovation at Harbor-UCLA Medical Center, Torrance, California; 8 David Geffen School of Medicine, University of California, Los Angeles; 9 Department of Human Ecology, College of Agricultural and Environmental Sciences, University of California, Davis; 10 California Preterm Birth Initiative, University of California, San Francisco; 11 Department of Rheumatology, University of California, San Francisco; 12 Department of Public Health, College of Health and Human Services, California State University, Fresno

## Abstract

**Importance:**

Racial, ethnic, and income disparities in perinatal depression prevalence and treatment are partially driven by social determinants of health. Effective treatments addressing these determinants are needed.

**Objective:**

To determine whether enhanced group prenatal care (eGPC) outperforms enhanced individual prenatal care (eIPC) for reducing perinatal depressive symptoms.

**Design, Setting, and Participants:**

This randomized clinical trial was conduced in 10 Medicaid-serving clinics in California’s San Joaquin Valley, enrolling English- or Spanish-speaking Medicaid-eligible pregnant individuals at less than 25 weeks’ gestation, from November 2019 to January 2024, with 2 follow-up surveys through 12 weeks postpartum. Analyses were conducted as intention-to-treat. Data were analyzed from December 2024 to December 2025.

**Interventions:**

Participants were randomized to eIPC or eGPC. eIPC enhancements included assessments tailored to individual psychosocial, clinical, oral health, and substance use needs. eGPC enhancements included childcare, perinatal mental health screening and referral, transportation stipends, free groceries, and information on community resources.

**Main Outcomes and Measures:**

The primary outcome was depression, operationalized as change in Patient Health Questionnaire–9 scores from baseline to 3 months postpartum. Outcomes were assessed by masked assessors.

**Results:**

Of 1663 individuals assessed, 678 were enrolled and randomized; 4 withdrew consent, yielding an analyzed sample of 674 participants (mean [SD] age, 27.0 [5.8] years), including 50 African American or Black participants (7.4%); 37 biracial, multiracial, or multiethnic participants (5.5%); 485 Latine participants (72.0%); 77 White participants (11.4%); and 24 participants who identified as another race or ethnicity (3.6%). After randomization, there were 294 participants in the eGPC group and 380 participants in the eIPC group. No difference in reductions in depressive symptom severity from baseline to 3 months postpartum by randomization group was observed (Cohen *d* for between-group change, 0.1; 95% CI, –0.1 to 0.3; *P* = .45), adjusting for baseline depressive symptom severity, self-reported history of a mental health condition, language, and calendar time at enrollment. Instead, participants in both groups experienced small to moderate reductions in depression symptoms from baseline to 3 months postpartum (eGPC: mean [SD] difference, −2.2 [5.3]; Cohen *d* = −0.4; 95% CI, −0.6 to −0.3; *P* < .001; eIPC: mean [SD] difference, −1.6 [4.5]; Cohen *d* = −0.5; 95% CI, −0.6 to −0.4; *P* < .001).

**Conclusions and Relevance:**

In this randomized clinical trial of pregnant, low-income, primarily Latine individuals, statistically significant improvements were observed in depressive symptom severity from baseline to postpartum, regardless of prenatal care type. There was no evidence of a difference between enhanced prenatal care types for improving depressive symptoms.

**Trial Registration:**

ClinicalTrials.gov Identifier: NCT04154423

## Key Points

**Question:**

Does enhanced group prenatal care lead to greater reductions in depressive symptom severity relative to enhanced individual prenatal care among pregnant individuals with low incomes?

**Findings:**

In this randomized clinical trial including 674 individuals, reductions in depressive symptom severity from baseline to 3 months postpartum did not differ by enhanced prenatal care type. Participants in both prenatal care types reported statistically significant reductions in depressive symptom severity.

**Meaning:**

This randomized clinical trial did not find evidence of a difference between enhanced prenatal care types for improving depressive symptoms.

## Teaser

This randomized clinical trial examines the effects of enhanced group prenatal care vs enhanced individual prenatal care for postpartum depression among low-income pregnant individuals in California.

## Introduction

Depression during pregnancy or the postpartum period (ie, perinatal depression) is common, affecting approximately 12% of perinatal individuals.^1,2^ The consequences of untreated perinatal depression are severe and enduring, including increased risk of adverse birth outcomes,^3,4,5^ maternal suicide,^6^ and impairments in parenting.^7^ Untreated perinatal depression leads to societal costs totaling billions of dollars annually due to productivity loss, increased use of Medicaid, and higher health care expenditures.^8^ Furthermore, there are racial, ethnic, and income disparities in the prevalence and treatment of perinatal depression,^9,10,11^ which are shaped by structural and social drivers of health (SDOH). Factors such as food insecurity, housing instability, low social support, and transportation challenges are consistently associated with elevated perinatal depression symptoms.^12,13,14,15^ Whereas only approximately half of depressed pregnant individuals receive treatment,^16,17,18^ most pregnant individuals receive prenatal care. Thus, targeting the factors that contribute to perinatal depression within routine prenatal care represents a promising strategy for addressing unmet needs.

Group prenatal care (GPC) delivers guideline-consistent prenatal care in monthly groups of up to 12 pregnant individuals at approximately the same gestational age.^19^ It provides structured peer support, psychoeducation, and structured coping activities, which directly address several mechanisms underlying depression improvement (eg, social support, behavioral activation). Group care can be further enhanced to include sexual risk reduction content, cognitive behavioral therapy, or linkage to other social services. The evidence regarding the effects of GPC on mental health outcomes is mixed. One study among pregnant adolescents found significant overall improvements in depressive symptoms when comparing an enhanced GPC (eGPC) model to individual prenatal care.^20^ In contrast, 4 studies found no significant differences between care types.^21,22,23,24^ However, 2 additional studies observed beneficial effects specifically in the subgroups of individuals experiencing higher psychosocial risk, including 1 study that tested standard GPC and 1 that tested an enhanced version.^25,26^

Given national recommendations for scalable, integrated mental health solutions in primary care,^27^ as well as calls for larger well-designed studies to investigate the impact of GPC on mental health,^28^ the Engaging Mothers and Babies–Reimagining Antenatal Care for Everyone (EMBRACE) study evaluated whether enhanced prenatal care models can reduce depressive and anxiety symptoms within clinical settings.

Our primary aim was to compare the effect of eGPC vs eIPC on changes in depressive symptom severity (primary outcome; Patient Health Questionnaire 9-item [PHQ-9]^29^) and anxiety symptom severity (secondary outcome; Generalized Anxiety Disorder 7-item [GAD-7] scale^30^) from baseline (<25 weeks’ gestation) to 3 months postpartum. We hypothesized that participants assigned to eGPC would experience greater reductions in depressive and anxiety symptom severity from baseline to 3 months postpartum (primary time point) and in the third trimester (secondary time point) compared with participants assigned to eIPC. We anticipated eGPC would outperform eIPC because (1) GPC addresses mechanisms known to improve depression (eg, social support, behavioral activation); (2) eGPC incorporates mental health augmentations, including psychoeducation about perinatal depression, strategies for managing worry and improving sleep, and symptom screening and facilitated referral to a perinatal mental health team; and (3) eGPC addresses SDOH-related stressors linked to perinatal depression (eg, food insecurity, low social support, childcare constraints, transportation barriers^12,13,14,15^) by providing groceries, transportation, and childcare. Although eIPC also addressed SDOH, eGPC offered a more comprehensive set of supports and a delivery format that targets multiple depression-related mechanisms concurrently.

## Methods

### Study Design

The goals of the EMBRACE study were to compare the effects of eGPC and eIPC on mental health (primary outcome), the experience of care (secondary outcome), and preterm birth (exploratory outcome). The EMBRACE Study protocol and statistical analysis plan have been published previously^1^ and are provided in Supplement 1. Institutional review boards at participating institutions approved the study. Participants provided written informed consent prior to enrollment. This study is reported following the Consolidated Standards of Reporting Trials (CONSORT) reporting guideline.

### Participants

Eligible participants were English- or Spanish-speaking pregnant individuals with incomes at or below 213% of the federal poverty level (ie, Medicaid eligible), recruited before 25 weeks’ gestation from 10 Medicaid-serving clinics staffed by multidisciplinary prenatal care practitioners in California’s San Joaquin Valley (Fresno, Merced, and Kern Counties). Race and ethnicity were self-reported and categorized African American or Black, biracial or multiracial, Latine, White, and other race or ethnicity (eg, American Indian, Alaska Native, or Indigenous; Asian; or Native Hawaiian or Pacific Islander). Further details are provided in the eMethods in Supplement 2.

### Randomization and Masking

The randomization scheme was generated by the study statistician (C.E.M.), who was the only individual with access to the allocation sequence. When a new practitioner was ready to be randomized, study staff contacted the study statistician, who then revealed the assignment. Further details are available in the trial protocol in Supplement 1. Randomization was stratified by prenatal care practitioner. Clinics offered both types of enhanced prenatal care, and practitioners alternated between intervention strategies in 6-week blocks after being randomly allocated to a starting strategy. To promote group cohesion and ensure that participants received a sufficient dose of eGPC, groups were closed to enrollment after session 4. In contrast, eIPC had no such operational constraints and allowed rolling enrollment for the duration of the 6-week enrollment period. Enrollment occurred from November 2019 to January 2024, with 2 follow-up surveys through 12 weeks postpartum. Participants received remuneration for completing each questionnaire ($30 baseline, $50 third trimester, and $50 postpartum).

To ensure group assignments were masked from primary- and secondary-outcome assessors, research staff who conducted baseline interviews were different from those who conducted third trimester and postpartum interviews.

### Interventions

The EMBRACE study was a randomized clinical trial comparing eGPC and enhanced individual prenatal care (eIPC), both of which were enhanced to address SDOH. Development of the eGPC model was informed by pilot-phase focus group feedback; it was further augmented to include psychoeducation about perinatal depression, brief strategies for managing worry and improving sleep, and depressive symptom screening and referral to a perinatal mental health team for individuals with positive screening results. We conducted the study with a sample at heightened risk of perinatal depression: predominantly Latine individuals with low income.^9,10,11^

The Comprehensive Perinatal Services Program is a California Medicaid benefit offering culturally attentive, billable eIPC support services provided by approved community health workers, health educators, nurses, or prenatal care practitioners. Services include an initial health assessment and education, followed by 3 additional assessments tailored to each individual’s health-related social needs (eg, psychosocial, clinical, oral health, and substance use). These services are offered with individual prenatal care.

eGPC was community-developed and includes wrap-around services and enhancements to address SDOH, including childcare, perinatal mental health screening and referral, transportation stipends, free groceries, and information on community resources. Enhancements addressing SDOH were delivered by First 5 Fresno County, a governmental agency charged with implementing an integrated system of services to support the early development of children from the prenatal stage to age 5 years. First 5 Fresno County provided oversight, training, and contracted staff facilitators to coordinate the delivery of wraparound services and cofacilitate sessions with practitioners. For the eGPC component, 11 prenatal care practitioners and 7 staff facilitators trained in the CenteringPregnancy curriculum^19^ led 7 to 10 English, Spanish, or bilingual sessions with up to 12 patients who had estimated delivery dates within a 6-week range.

Both eGPC and eIPC expand on the core elements of their standard counterparts, offering Medicaid-eligible pregnant people more comprehensive and integrated care. Compared with IPC, the eIPC model adds comprehensive psychosocial assessment, structured SDOH screening, individualized care plans, and coordinated referrals to routine clinical prenatal care. Similarly, eGPC builds on GPC (the CenteringPregnancy curriculum) by adding mindfulness activities; structured mental health screening at every session; wraparound support to address social needs, such as transportation, childcare, and food support; and informational support on community resources to mitigate SDOH. Further detail about the unique and common features across prenatal care delivery models and enhancements are provided in eTable 1 in Supplement 2. Details about how eGPC and eIPC were adapted during the COVID-19 pandemic are provided in the eMethods in Supplement 2.

### Outcomes

The primary outcome for EMBRACE was depression, operationalized as the change in PHQ-9 score from baseline to 3 months postpartum and, secondarily, from baseline to the third trimester.^29^ PHQ-9 scores range from 0 to 27, with scores of 10 or greater indicating moderate to severe depression.^29^ PHQ-9 is frequently used in clinical practice and has been validated among perinatal samples, with sensitivity and specificity rates of 85% and 84%, respectively.^10^ Scores do not significantly vary by interviewer vs self administration.^31^ We used the GAD-7 scale to assess change in anxiety symptom severity from baseline to 3 months postpartum and baseline to the third trimester.^32^ GAD-7 scores range from 0 to 21; among pregnant and postpartum individuals, a cutoff of 13 has sensitivity of 61% and specificity of 73% for detecting diagnoses of generalized anxiety disorder.^30^ Two other outcomes were measured to gain a nuanced understanding of participants’ mental well-being. To assess subjective stress, we used the Perceived Stress Scale, a 10-item self-report measure with total scores ranging from 0 to 40, with higher scores indicating greater stress levels.^33^ The Perceived Stress Scale has been validated in perinatal samples.^34^ We used the Patient-Reported Outcome Measurement Information System Sleep Disturbance–Short Form 8b to measure sleep.^35,36^ Total scores were converted to *t* scores. All of these outcome assessments were verbally administered by study staff.

### Sample Size Calculation

Sample size was determined to guarantee adequate power for the primary outcome of depression. Our primary hypothesis was that participants randomized to eGPC would have larger reductions in depressive symptom severity than those randomized to eIPC at 3 months postpartum compared with baseline. Consistent with existing literature, our sample size calculations were based on an effect size of 0.25, with a pooled SD of 4.9 for the PHQ-9 change score from baseline to 3 months postpartum. This is equivalent to an estimated absolute effect size (difference in PHQ-9 change score) of 1.2 points.^37^ Assuming a similar retention rate for the postpartum interview that we achieved in our pilot study (88%), we calculated that enrolling 657 participants would yield 85% power for our primary analysis.

### Statistical Analysis

For our primary and secondary outcomes, changes in depressive and anxiety symptom severity from baseline to third trimester and from baseline to 3 months postpartum were compared using linear models adjusted for baseline value of the outcome, self-reported history of mental health condition, language of interview, and calendar time at enrollment as a restricted cubic spline (with 4 knots). Generalized estimating equations methods were used with cluster robust standard errors at the practitioner level.

Missing data were addressed using multiple imputation (100 imputations using Markov chain Monte Carlo). To address issues of nonnormality, restricted range scales were transformed prior to imputation and then back-transformed.^38^ Variables needed to categorize subsets (eg, race and ethnicity), were imputed using chained equations with the appropriate distributional type. Details about sensitivity analyses and heterogeneity of treatment effects analyses are provided in the eMethods in Supplement 2.

*P* values were 2-sided, and statistical significance was set at *P* < .05. Statistical analyses were conducted using SAS software version 9.4 (SAS Institute) and Stata software version 19 (StataCorp) from December 2024 to December 2025.

## Results

### Baseline Patient Characteristics and Participant Flow

Of 1663 individuals screened for eligibility, 678 met criteria, were enrolled between November 2019 and January 2024, and were randomly assigned to eGPC (298 individuals) or eIPC (380 individuals). Four participants withdrew consent, yielding an analytic sample of 674 participants (mean [SD] age, 27.0 [5.8] years), including 294 participants in the eGPC group and 380 participants in the eIPC group (Figure). Data collection occurred between November 2019 and December 2024.

**Figure.**
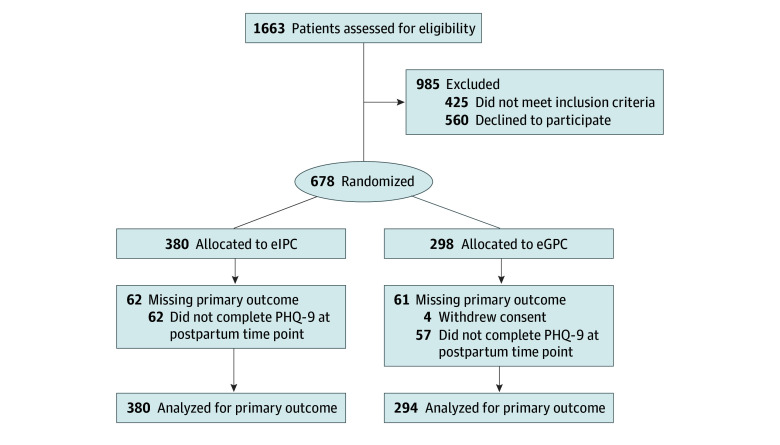
CONSORT Diagram of Participant Flow Through Trial The diagram displays the number of participants who were assessed for eligibility, randomized, contributed follow-up data, and were included in analyses. Missing data were handled with multiple imputation. CONSORT indicates Consolidated Standards of Reporting Trials; eGPC, enhanced group prenatal care; eIPC, enhanced individual prenatal care; and PHQ-9, Patient Health Questionnaire.

Follow-up PHQ-9 data were available for 544 participants (80.7%) at the third trimester and 555 participants (82.3%) at 3 months postpartum (primary end point). Most participants were aged at least 25 years (471 participants [61.9%]), and married or living with a partner (503 participants [74.6%]) (Table 1). There were 50 African American or Black participants (7.4%); 37 biracial, multiracial, or multiethnic participants (5.5%); 485 Latine participants (72.0%); 77 White participants (11.4%); and 24 participants who identified as another race or ethnicity (3.6%). A total of 188 participants (27.9%) were born in Latin America, and 125 participants (18.5%) completed surveys in Spanish. Approximately 1 in 5 participants reported a lifetime diagnosis of depression (154 participants [22.8%]) or anxiety (139 participants [20.6%]). Overall, 114 participants (16.9%), 68 participants (10.1%), and 53 participants (7.9%) scored above the cutoff on the PHQ-9 at baseline, third trimester, and 3 months postpartum, respectively.

**Table 1.** Participant Sociodemographic and Clinical Characteristics

Characteristic	Participants, No. (%)^a^		
	Total (N = 674)	eGPC (n = 294)	eIPC (n = 380)
Age, mean (SD), y	27.0 (5.8)	26.5 (5.6)	27.4 (6.0)
Race and ethnicity			
African American or Black	50 (7.4)	21 (7.1)	29 (7.6)
Biracial, multiracial, or multiethnic^b^	37 (5.5)	18 (6.1)	19 (5.0)
Latine^c^	485 (72.0)	203 (69.0)	282 (74.2)
White	77 (11.4)	41 (13.9)	36 (9.5)
Other^d^	24 (3.6)	11 (3.7)	13 (3.4)
Birthplace			
United States	474 (70.3)	217 (73.8)	257 (67.6)
Latin America^e^	188 (27.9)	70 (23.8)	118 (31.1)
Other^f^	10 (1.5)	6 (2.0)	4 (1.1)
Opted for Spanish-language version of study	125 (18.5)	46 (15.6)	79 (20.8)
Education			
<High school graduate	152 (22.6)	66 (22.4)	86 (22.6)
High school graduate or equivalent	247 (36.6)	107 (36.4)	140 (36.8)
Some college or vocational school	207 (30.7)	93 (31.6)	114 (30.0)
≥4-y college degree	67 (9.9)	27 (9.2)	40 (10.5)
Monthly household income, $			
<1000	154 (22.8)	58 (19.7)	96 (25.3)
1000-2000	202 (30.0)	79 (26.9)	123 (32.4)
2001-3000	154 (22.8)	72 (24.5)	82 (21.6)
>3000	130 (19.3)	67 (22.8)	63 (16.6)
Do not know	26 (3.9)	14 (4.8)	12 (3.2)
Relationship status			
Married, living with partner	503 (74.6)	218 (74.1)	285 (75.0)
Partnered, not living together	99 (14.7)	42 (14.3)	57 (15.0)
Single	72 (10.7)	34 (11.6)	38 (10.0)
Parity			
0	206 (30.6)	99 (33.7)	107 (28.2)
1-2	304 (45.1)	128 (43.5)	176 (46.3)
≥3	163 (24.2)	67 (22.8)	96 (25.3)
Self-reported lifetime diagnosis of mental health condition^g^			
Depression	154 (22.8)	71 (24.1)	83 (21.8)
Anxiety	139 (20.6)	67 (22.8)	72 (18.9)
Other or unspecified	42 (6.2)	17 (5.8)	25 (6.6)

Abbreviations: eGPC, enhanced group prenatal care; eIPC, enhanced individual prenatal care.

^a^ Percentages may not add to 100 due to missing data.

^b^ Includes Latine and White (14 individuals); Black and Latine (4 individuals); Black and White (4 individuals); Latine and Native Hawaiian or Pacific Islander (2 individuals); Latine, Native American, American Indian, Alaska Native, or Indigenous, and White (2 individuals); Asian and Latine (1 individual); Latine and Native American, American Indian, Alaska Native, or Indigenous (1 individual); White and Asian (1 individual); Native American, American Indian, Alaska Native, or Indigenous and Black (1 individual); Black, Latine, and White (1 individual); Asian, Black, and Latine (1 individual); Native American, American Indian, Alaska Native, or Indigenous, Black, Latine, and White (1 individual); Native American, American Indian, Alaska Native, or Indigenous, Latine, Native Hawaiian or Pacific Islander, and White (1 individual); Native American, American Indian, Alaska Native, or Indigenous, Black, Latine, and Native Hawaiian or Pacific Islander (1 individual); Native American, American Indian, Alaska Native, or Indigenous, Black, Native Hawaiian or Pacific Islander, and White (1 individual).

^c^ Includes Latina, Latinx, or Hispanic.

^d^ Includes Asian (18 individuals); Native American, American Indian, Alaskan Native, or Indigenous (3 individuals); and Pacific Islander or Native Hawaiian (3 individuals).

^e^ Includes Mexico (173 individuals), El Salvador (7 individuals), Guatemala (4 individuals), Honduras (2 individuals), Dominican Republic (1 individual), Nicaragua (1 individual).

^f^ Includes Philippines (3 individuals), India (2 individuals), Armenia (1 individual), China (1 individual), Egypt (1 individual), Thailand (1 individual), Yemen (1 individual).

^g^ These are the numbers of people who replied yes to the question “[H]ave you ever been diagnosed with or treated for a mental health condition, such as depression, anxiety, or something else?”

### Primary and Secondary Outcomes

Contrary to hypotheses, participants assigned to eGPC did not experience greater reductions in depressive symptom severity from baseline to 3 months postpartum compared with participants assigned to eIPC (Cohen *d* for between-group change, 0.1; 95% CI, –0.1 to 0.3; *P* = .45), adjusting for baseline depressive symptom severity, self-reported history of a mental health condition, language, and calendar time (Table 2). Instead, participants in both groups experienced small to moderate reductions in depression symptoms from baseline to 3 months postpartum (eGPC: unadjusted mean [SD] difference, −2.2 [5.3]; adjusted Cohen *d* = −0.4; 95% CI, −0.6 to −0.3; *P* < .001; eIPC: unadjusted mean [SD] difference, −1.6 [4.5]; adjusted Cohen *d* = −0.5; 95% CI, −0.6 to −0.4; *P* < .001). Baseline to third trimester analyses yielded the same pattern of findings (Table 2).

**Table 2.** Primary and Secondary Outcome Measures With Within-Group and Between-Group Effect Sizes (N = 674)

Outcome	Imputed mean (SD)			Within-group standardized mean difference (95% CI)^a^^,^^b^		Between-group standardized mean difference (95% CI)^a^^,^^c^	
	Baseline	Third trimester	Postpartum	Baseline to third trimester	Baseline to postpartum	Baseline to third trimester	Baseline to postpartum
**PHQ-9**								
eGPC	6.1 (4.8)	5.4 (4.4)	3.9 (4.4)	−0.1 (−0.2 to 0.0)	−0.4 (−0.6 to −0.3)^d^	0.2 (−0.0 to 0.3)	0.1 (−0.1 to 0.3)
eIPC	4.7 (4.0)	4.2 (4.0)	3.1 (4.1)	−0.2 (−0.3 to −0.1)^d^	−0.5 (−0.6 to −0.4)^e^		
**GAD-7**								
eGPC	4.5 (4.7)	4.3 (4.4)	3.6 (4.5)	0.0 (−0.1 to 0.2)	−0.1 (−0.3 to 0.0)	0.1 (−0.1 to 0.3)	0.1 (−0.1 to 0.3)
eIPC	3.4 (3.9)	3.4 (3.9)	2.9 (4.2)	−0.1 (−0.2 to 0.0)	−0.2 (−0.3 to −0.1)^e^		

Abbreviations: eGPC, enhanced group prenatal care; eIPC, enhanced individual prenatal care; GAD-7, Generalized Anxiety Disorder Scale; PHQ-9, Patient Health Questionnaire-9.

^a^ Negative effect sizes indicate a reduction in symptoms.

^b^ Estimated standardized change in mean values over time within a group, adjusted for baseline value of outcome, self-reported history of a mental health condition, calendar time at enrollment (as a restricted cubic spline with 4 knots), and language of the questionnaire.

^c^ Standardized difference between groups in estimated change over time in mean values, adjusted for baseline value of outcome, self-reported history of a mental health condition, calendar time at enrollment (as a restricted cubic spline with 4 knots), and language of the questionnaire.

^d^ *P* < .001.

^e^ *P* < .01.

Participants assigned to eGPC also did not experience greater reductions in anxiety symptom severity compared with participants assigned to eIPC (baseline to third trimester Cohen *d* = 0.1; 95% CI, −0.1 to 0.3; baseline to 3 months postpartum Cohen *d* = 0.1; 95% CI, −0.1 to 0.3). Findings from the sensitivity analyses and heterogeneity of treatment effect analyses are presented in the eResults in Supplement 2.

## Discussion

In this randomized clinical trial, we found that eGPC did not outperform eIPC for improving mental health outcomes in this sample of primarily Latine pregnant individuals with low incomes. Participants experienced statistically significant improvements in depressive symptom severity and stress from baseline to 3 months postpartum, regardless of prenatal care type. These findings are consistent with research by Gennaro et al^24^ comparing 2 eGPC programs in a sample of Black and Hispanic participants with elevated depression, anxiety, or stress scores. Enhancements in the study by Gennaro et al^24^ included either a 6-session cognitive behavioral therapy protocol or 6-session attention-matched control with relevant pregnancy education. Across care types, participants reported statistically significant improvements in depression, anxiety, and stress.^24^ In our study, effect sizes for depression outcomes were large in the subgroup of participants with elevated baseline depression scores, suggesting that enhancing prenatal care, regardless of whether the care is provided individually or in a group, may be particularly beneficial for this population. Heterogeneity of treatment effect analyses suggested that prenatal care type (group or individual) did not have differential effects on mental health outcomes across racial, ethnic, or depression risk subgroups.

The relatively low levels of depression and anxiety reported by this historically marginalized sample during the COVID-19 pandemic was unexpected. In our study, 16.9%, 10.1%, and 7.9% of participants scored above the cutoff on the PHQ-9 at baseline, third trimester, and postpartum, respectively. These rates are lower than prior reports among perinatal individuals with low income and during the COVID-19 pandemic.^39^ A meta-analysis of 54 studies indicated that the prevalence of depression increased during the pandemic, with rates as high as 31.4% and 27.6% among pregnant and postpartum individuals, respectively.^40^ Unexpectedly low prevalence rates in our sample may reflect resilience factors, such as strong social support,^41^ which could have buffered against psychological distress, but future research is needed to investigate this hypothesis. Additionally, these findings may be related to the significant representation of Latine pregnant individuals in our study population. For example, a nationally representative sample of pregnant people found that Latine ethnicity was associated with a reduced likelihood of postpartum mental health conditions.^42^ Lower levels of acculturation have also been linked to decreased rates of perinatal mental health disorders, with the hypothesis that traditional cultural practices may offer a buffering effect against stress and social disadvantage.^43,44^ While acculturation was not explicitly considered in our analysis, the study population (27.9% born in Latin America and 18.5% completing surveys in Spanish) may exhibit lower levels of acculturation, which could contribute to the unexpectedly low prevalence rates. Alternatively, as suggested by local stakeholders during a presentation of preliminary findings, participants may have underreported depression symptoms in the interviewer-administered PHQ-9 due to mental health stigma^45^ or social desirability bias. Consistent with this, prior research indicates that stigma can be a powerful barrier to disclosing and seeking treatment for perinatal depression.^45,46,47,48^ Finally, prior research has documented more variability in cutoff PHQ-9 scores for detecting major depressive disorder among Spanish-speaking participants, perhaps indicating cultural differences in symptom detection.^49^

Even subthreshold symptoms of perinatal depression are associated with significant impairments in functioning.^50^ Thus, it is especially encouraging that participants in our study experienced statistically significant symptom improvements, despite being at heightened risk for symptom exacerbation due to sociodemographic factors. Although participants did not meet the thresholds for minimal clinically important differences on the PHQ-9 (ie, −2.0) or the GAD-7 (ie, −2.2).^37^ Nevertheless, even very small effect sizes can yield substantial health benefits when implemented at the population level.^51^

### Strengths and Limitations

This study has some strengths, as well as some limitations. Strengths of this study include implementation in clinical settings, stakeholder involvement in both study design and execution, and high participant retention. Our focus on this sample of predominantly Latine pregnant individuals with low incomes in California’s San Joaquin Valley is a key strength, although it may limit generalizability to other perinatal populations. Moreover, most of the study was conducted during the COVID-19 pandemic, which required shifting delivery of eGPC from in-person to telehealth. It is possible telehealth delivery impacted the social support and community provided by eGPC, thus attenuating mental health benefits. Attendance at eGPC sessions was lower than expected, and many cohorts were cancelled if enrollment was fewer than 4 participants by the fourth session or due to practitioner illness, resulting in reduced exposure to the mental health enhancements. As a result, it remains unclear whether null findings reflect limited efficacy of eGPC or suboptimal implementation. Substantially fewer participants were allocated to the eGPC group because groups were closed after session 4, whereas eIPC had rolling enrollment for the entire 6-week enrollment period. In addition, because practitioners delivered both IPC and GPC, contamination of practitioner behaviors is possible, which could attenuate between-group differences. However, several features of the interventions were less susceptible to contamination (eg, group vs individual delivery, nonclinical cofacilitator in eGPC). Furthermore, we do not have data on utilization of mental health treatments, limiting our ability to assess potential differential treatment utilization across care types.

## Conclusions

The findings of this randomized clinical trial add to the evolving evidence that enhancing prenatal care, whether delivered in group or individual formats, can support meaningful improvements in perinatal mental health among populations with low income. Although eGPC was not superior to eIPC, the observed symptom reductions across both conditions suggest that embedding brief, scalable mental health supports into routine prenatal care holds promise for addressing mental health disparities during the perinatal period. These findings can inform decision-making for patients and practitioners, especially in settings with constraints on time, staffing, or access to specialized mental health services. Future research should investigate which specific components of enhanced prenatal care (eg, psychoeducation, peer support, increased time and attention from clinicians) are most critical.

## Notes

Supplement 1.
Trial Protocol and Statistical Analysis Plan

Supplement 2.
eMethods.

eResults.
**eTable 1.** Unique and common features across prenatal care delivery models and enhancements. **eTable 2.** Imputed means (standard deviations), within-group effect sizes, and between-group effect sizes in the Latine and Black subgroups **eTable 3.** Imputed means (standard deviations), within-group effect sizes, and between-group effect sizes in the subgroups with elevated depression risk **eTable 4.** Imputed means (standard deviations), within-group effect sizes, and between-group effect sizes for exploratory stress and sleep outcomes (n=674)

Supplement 3.
Data Sharing Statement
