# Supplementary material for: Enhanced Prenatal Care Models and Postpartum Depression: The EMBRACE Randomized Clinical Trial
Source: JAMA Netw Open. 2026 Feb 16;9(2):e2559883. doi: 10.1001/jamanetworkopen.2025.59883 (PMC12910397; doi:10.1001/jamanetworkopen.2025.59883)
Supplement: Supplement 1. — Trial Protocol and Statistical Analysis Plan [file jamanetwopen-e2559883-s001.pdf]

Comparing Approaches to Enhanced Prenatal Care to Improve Maternal and Child Health in Central CA

Engaging Mothers & Babies – Reimagining Antenatal Care for Everyone (EMBRACE) Study

Sponsored by:
Patient-Centered Outcomes Research Institute (PCORI)

Contract Number AD-2018C2-13227
September 1, 2019 – March 31, 2026
Protocol Date: August 23, 2023

IRB Approvals
UCSF IRB# 19-28319
CSU Fresno IRB# 922

Principal Investigator: Miriam Kuppermann, PhD, MPH
Professor, Departments of Obstetrics, Gynecology & Reproductive Sciences and Epidemiology & Biostatistics
Director, Prenatal Interventions, California Preterm Birth Initiative
University of California, San Francisco

The research described in this protocol was funded through a Patient-Centered Outcomes Research Institute (PCORI) Award [AD-2018C2-13227]. The statements in this protocol are solely the responsibility of the authors and do not necessarily represent the views of the Patient-Centered Outcomes Research Institute (PCORI), its Board of Governors, or Methodology Committee. The funder had no role in study design; collection, management, analysis, or interpretation of data; writing of the report; and the decision to submit findings for publication.

TABLE OF CONTENTS

Study Team Roster 4

Personnel and Duties 5

Study Schema 8

1.0 Introduction 9

1.1 Background and Prior Research 9

1.1.1 Conceptual Framework 12

2.0 Study Hypothesis and Design 13

2.1 Hypotheses 13

2.2 Study Design 14

Figure: Consort Diagram 15

2.3 Randomization 14

Figure: Example Provider Recruitment Track with Randomization 16

3.0 Study Population 16

3.1 Inclusion Criteria 16

3.2 Exclusion Criteria 16

3.3 Participant Retention 17

3.4 Participant Withdrawal 17

4.0 Study Arms 17

4.1 Glow/GC 17

4.2 CPSP/IC 18

5.0 Study Procedures 18

5.1 Overview 18

5.1.1 Prenatal Care Medical Record Screening 20

5.1.2 Screening and Approach 20

5.1.3 Enrollment and Baseline Interview 19

5.1.4 Third Trimester Follow-up Interview 21

5.1.5 Medical Record Abstraction 22

5.1.6 Postpartum Interview 22

5.2 Timeline 23

6.0 Saftey Monitoring and Adverse Event Reporting 23

6.1 Saftey Monitoring and Clinical Data Review 23

6.2 Reporting Requirments for this Study 24

6.3 Interim Analysis and Data Oversight 25
7.0 Statistical Considerations....................25
7.1 Endpoints 25
7.1.1 Primary Endpoint Aim 1 25
7.1.2 Secondary Endpoints Aim 1 25
7.1.3 Primary Endpoint Aim 2 25
7.1.4 Secondary Endpoints Aim 2....................25
7.1.5 Primary Endpoint Aim 3 26
7.1.6 Secondary Endpoints Aim 3....................26
7.2 Sample Size 26
7.3 Inclusion of Women and Minorities 26
7.4 Masking....................26
7.5 Data Analysis 27
8.0 Human Subjects Considerations....................29
8.1 Ethical Review....................29
8.2 Informed Consent 29
8.3 HIPAA....................29
8.4 Risks....................29
8.5 Benefits....................30
8.6 Incentives....................30
8.6 Confidentiality 30
9.0 Administrative Procedures 30
9.1 Study Coordination 30
9.1.1 Trainings....................30
9.1.2 Study Communicaton 31
9.2 Database Management and Data Quality Mointoring 32
9.3 Use of Information and Publications....................33
10.0 Important Changes to Original Protocol....................33
11.0 References 34

STUDY TEAM ROSTER

**University of California, San Francisco (UCSF)**		
**Investigators**		
Miriam Kuppermann, PhD, MPH	Principal Investigator	Miriam.Kuppermann@ucsf.edu
Patience Afulani, MBChB, MPH, PhD	Co-Investigator, Measurement	Patience.Afulani@ucsf.edu
Bridgette Blebu, PhD, MPH	Co-Investigator, Implementation Science	Bridgette.Blebu@ucsf.edu
Brittany Chambers, PhD, MPH	Co-Investigator, Qualitative Lead	Brittany.Chambers@ucsf.edu
Kimberly Coleman-Phox, MPH	Co-Investigator, Study Implementation	Kimberly.Coleman-Phox@ucsf.edu
Christopher Downer, MD	Co-Investigator, Obstetrics and Gynecology, UCSF Fresno	Christopher.Downer@ucsf.edu
Jennifer Fielder, PhD	Co-Investigator, Psychology	Jennifer.Felder@ucsf.edu
Deborah Karasek, PhD, MPH	Co-Investigator, Epidemiology	Deborah.Karasek@ucsf.edu
Daisy Leon-Martinez, MD	Co-Investigator, Maternal Fetal Medicine	Daisy.Leon-Martinez@ucsf.edu
Charles McCulloch, PhD	Co-Investigator, Biostatistics	Charles.McCulloch@ucsf.edu
Martha Tesfalul, MD	Co-Investigator, Maternal Fetal Medicine	Martha.Tesfaul@ucsf.edu
**Staff**		
Cinthia Blat, MPH	Statistical Analyst	Cinthia.blat@ucsf.edu
Eniola Owoyele	Medical Record Abstractor	Eniola.Owoyele@ucsf.edu
Bethany Simard, MPH	Data Manager	Bethany.Simard@ucsf.edu
**California State University, Fresno (CSU Fresno)**		
**Investigators**		
Venise Curry, MD	Co-Investigator, Director of Community Engagement	Vccurry24@gmail.com
Mary A. Garza, PhD, MPH	Co-Investigator, Site Director	Magarza@csufresno.edu
Lauren Lessard, PhD, MPH	Co-Investigator, Policy	lnlessard@alaska.edu

Staff		
Lupita Hernadez-Rojo	Recruitment and Retention Specialist	lupitaaahr@gmail.com
Geraldine Alvarez	Recruitment and Retention Specialist	ideanj0810@mail.fresnostate.edu
Kesia Garibay	Recruitment and Retention Specialist	Kgaribay@mail.fresnostate.edu
Erica Martinez	Recruitment and Retention Coordinator	Erica04@csufresno.edu
Cierra Sanderson	Project Specialist	Cierra_sanderson@csufresno.edu
Kristin Carraway, MPH	Project Manager	Kristinc@csufresno.edu

PERSONNEL AND DUTIES

UCSF Study Team

Principal Investigator

As Principal Investigator for the EMBRACE Study, Dr. Miriam Kuppermann is responsible for all aspects of the design, conduct, and oversight of the study. This includes directing day-to-day activities, providing overall governance and scientific leadership to the study co-investigators and staff members, and taking the lead role in ensuring timely and accurate completion of the project and dissemination of findings. Dr. Kuppermann also has primary responsibility for ensuring that all milestones involving the development of study protocols and data collection tools, completion of data analytic tasks, and reporting requirements are met; that the study processes adhere to standards for rigorous research; and that the findings are widely disseminated. In addition, she is responsible for hiring and overseeing the UCSF research team.

Co-Investigators

All of the UCSF co-investigators contribute to the overall study design and execution, bringing the perspectives of the various disciplines they represent. They participate in monthly calls and in-person meetings as scheduled by the PI to provide input into study activities as needed. They will participate in refining the study design; developing study instrumentation; overseeing and conducting data analysis and interpretation; and preparing manuscripts for submission to peer-reviewed journals.

Data Manager

The data manager is responsible for creating and managing all databases for the project, monitoring data quality, and conducting data cleaning and statistical analyses under the direction of the PI, faculty biostatistician, and other co-investigators. She is in charge of creating monthly recruitment and retention reports for the funding agency.

Statistical Analyst

The statistical analyst is responsible for all multiple imputation tasks and higher level statistical analyses under the direction of the PI, faculty biostatistician, and other co-investigators.

Central Valley Health Policy Institute (CVHPI), California State University (CSU), Fresno Study Team

Site Director

Dr. Mary Garza, the CVHPI site director, is the primary administrative point of contact for California State University, Fresno. She is responsible for hiring and overseeing the CVHPI research team, ensuring that all recruitment and data collection milestones are met, and that the team adheres to the study protocol. She will attend investigator calls and in-person meetings as required by the PI and provide leadership and oversight to CVHPI team. She also will ensure that CVHPI research goals will be met in a timely manner, with integrity, within budgeted amounts, and within compliance regulations. The site director will be responsible for adverse event reporting to the PI.

Co-Investigator

The other CVHPI co-investigators also contribute to the overall study design and execution, bringing the perspectives of the various disciplines they represent.

Project Manager

The project manager is responsible for the overall operations of the CVHPI team's study activities and will manage study staff; implement study protocols; manage Fresno-specific research activities (including meeting enrollment and data collection milestones); and ensure that the study progress aligns with other milestones and contractual obligations. The project manager will report any potential revisions to protocols, surveys, and IRB approvals to the UCSF PI prior to their implementation. The project manager is the primary administrative point of contact for clinical practice coordination and will be responsive to provider needs. The project manager, with support from the PI and CVHPI team, is responsible for recruitment of clinical partners and developing and maintaining relationships with clinic staff and leadership to ensure recruitment goals can be met. The project manager will maintain a close relationship with First 5 Fresno County and the Fresno County Department of Public Health, the lead agencies for each comparator. The project manager will ensure that recruitment and retention staff work fluidly within practices and follow established IRB-approved recruitment procedures.

Project Specialist (PS)

The project specialist will assist the project manager with scheduling and agenda preparations for community and staff meetings, and other administrative duties as needed. She will maintain a close relationship with the Fresno County Department of Public Health, the lead agency for CPSP/IC comparator. She will coordinate payments to providers/sites and stipends to participants. The project specialist will assist with provider and participant recruitment as needed.

Recruitment and Retention Coordinator (RRC)

The recruitment and retention coordinator (RRC) will oversee screening, consent, allocation to comparator, and interviewing and remuneration of participants per protocol. The RRC will supervise the recruitment and retentions specialists (RRS, described below) throughout the study and be responsible for tracking recruitment status for each site and comparator. She will be responsible for following the guidelines for study operations outlined in this protocol and will promptly inform the project manager in the event of any protocol violations (e.g., mis-randomization, erroneous inclusion of subjects in the study, breach of confidentiality).

Recruitment and Retention Specialist (RRS)

The recruitment and retention specialists (RRS) are responsible for recruiting pregnant individuals into the study, interviewing participants at all study time points, and entering all collected data into the study database. They will be responsible for following the guidelines for study operations outlined in this protocol and will promptly inform the RRC in the event of any protocol violations (e.g., mis-randomization, erroneous inclusion of subjects in the study, breach of confidentiality).

Community Engagement Director and Co-investigator

The community engagement director and co-investigator will manage stakeholder engagement (Blue Ribbon Panel, Study Advisory Group, and Community Advisory Group) and coordinate community outreach to ensure that local residents will be involved in all aspects of the research design, implementation, and dissemination. They will coordinate with staff to bring together members from community initiatives focused on reducing disparate birth outcomes and racial disparities to ensure participants have positive interactions with systems of care, have reduced possibility of harm, and continue to collaborate on further improving prenatal care.

STUDY SCHEMA

TITLE: Engaging Mothers & Babies – Reimagining Antenatal Care for Everyone (EMBRACE)

Primary objectives: To determine whether pregnant individuals allocated to receive group prenatal care with wrap around services (Glow/GC; administered by First 5 Fresno County) versus pregnant individuals allocated to receive individual prenatal care with supplemental services covered by the California Department of Public Health Comprehensive Perinatal Services Program (CPSP/IC).

1. Have greater reductions in depressive symptom severity
2. Report more person-centered care
3. Have lower rates of preterm birth (exploratory)

Secondary objectives: To determine whether pregnant individuals allocated to Glow/GC versus CPSP/IC:

1. Have greater reductions in anxiety symptom severity
2. Report more person-centered maternity care, more respectful care, and greater satisfaction with prenatal care
3. Give birth at higher gestational ages

Design: Randomized comparative effectiveness study of two enhanced prenatal care programs.

Study population: English- or Spanish-speaking Medi-Cal eligible pregnant people receiving prenatal care at a participating healthcare facility in Fresno County.

Study comparators: Glow/GC, Comparator 1: Pregnant individuals participate in an enhanced group care model with 6-12 people during 8-11 telehealth or in-person sessions facilitated by a trained and licensed prenatal care practitioner and staff facilitator. Patients receive risk assessments, social support, prenatal care, and gain knowledge and skills related to pregnancy, birth and parenting. During the COVID-19 pandemic, this model has been modified to include virtual components as determined by the Glow/GC administrators and providers. This comparator is generically referred to as enhanced group prenatal care (eGPC).

CPSP/IC, Comparator 2: Pregnant individuals receive individual prenatal care and participate in CPSP, the state funded program for Medi-Cal eligible people, to receive enhanced care. Participants receive assessments with a Comprehensive Perinatal Health Worker at their prenatal care site. During the COVID-19 pandemic, some care is provided in person and some is provided via telehealth. This comparator is generically referred to as enhanced individual prenatal care (eIPC).

Study duration: Participants are enrolled during their first or second trimester of pregnancy and will be followed for up to 3 months postpartum, for a study duration of up to 10 months for each participant. Data collection began in November 2019 and was originally anticipated to end in March 2023 and will be completed by December 2024.

Sample size: 657 participants.

Study Sites:

1. Fresno ACC Family Health Care Network/UCSF Fresno Ob-Gyn Resident Clinic
2. St. Agnes Care Obstetrics and Gynecology
3. Clinica Sierra Vista-Elm Women's & Pediatric Community Health Center
4. Fresno Women's Medical Group
5. PineRidge Obstetrics and Gynecology

6. Adventist Health Medical Offices
7. Omni Women's Health Medical Group
8. Obria Medical Clinics of Central California,
9. United Health Centers-Mendota
10. Rio Bravo Family Medicine Residency Program
11. Timothy S. Johnston, MD and Associates

Additional sites to be added

1.0 INTRODUCTION

1.1 Background and Prior Research

Public health impact of preterm birth and racial inequity in the US. Approximately 10% of US births are preterm, occurring prior to 37 weeks gestation. Preterm birth (PTB) disproportionately affects low-income, Black, Latina, Pacific Islander, and Native American women.[1] PTB is serious and consequential: about 2/3 of infant deaths are among babies born preterm,[2] and survivors are at increased risk for physical, mental, and neurodevelopmental problems.[3] While clinical and social factors are known to be associated with PTB, the causes of observed socioeconomic and racial/ethnic disparities are poorly understood.[4] Perinatal depression and anxiety have been identified as important contributors to PTB risk, as have experiences of racism/discrimination, and disrespectful prenatal care; all are compounded by poverty.[5,6]

In Fresno County, which has the highest PTB rate in California, two enhanced prenatal care programs are available for Medi-Cal (California's Medicaid program) eligible pregnant individuals: the Department of Public Health's Comprehensive Perinatal Service Program (CPSP), and "Glow: Group Prenatal Care and Support." Glow was developed in response to community demand for solutions to address high rates of PTB and African American infant mortality, concerns about maternal mental health, and reports of negative interactions with prenatal care providers among low-income families.[7] Both programs focus on mitigating the effects of challenges related to social determinants of health, which contribute to disparities in pregnancy outcomes.

Despite its availability since 1984, rigorous studies of CPSP's effectiveness have not been conducted. And while some evidence indicates that group prenatal care may be effective in reducing PTB and improving maternal mental health,[8–12] whether it is more effective than individual care with CPSP services, or can result in more respectful care, is not known. As a result, low-income pregnant individuals do not have enough information to determine which enhanced prenatal care model would best serve their needs, and providers lack the evidence they need to make informed recommendations to patients.

Preterm birth rates remain stubbornly high, affecting roughly 10% of all US deliveries, with low income pregnant individuals bearing the highest burden.[1,13] Preterm birth has a substantial impact on the health of babies, mothers, and families: approximately 2/3 of all infant deaths occur among babies born prematurely,[14] and survivors are at increased risk for physical impairments, neurodevelopmental disabilities, poor cognitive functioning, and behavioral issues that may result in decreased educational attainment and lower lifetime earnings.[15,16] Moreover, pregnant individuals who were born preterm are at increased risk of delivering preterm.[17] Finally, along with the emotional distress families experience when caring for a preterm infant, the financial cost to individuals and society is substantial, estimated at an annual cost of $26.2 billion dollars in the US.[18]

While a number of mechanisms, including placental dysfunction, inflammation, and genetic factors, have been implicated in preterm birth,[4] much remains unknown about its causes, as most pregnant individuals who deliver early have no known risk factors.[4,19] Socioeconomic and racial/ethnic disparities, however, are well established.[20–22] For example, in 2010-2013 the preterm birth rate among Medi-Cal recipients was 12.7% compared to 10.9% among non-Medi-Cal recipients.[23] And while 9% of births to White women are preterm, 13.8% of Black, 11.4% of American Indian/Alaskan Native, 11.5% of Native Hawaiian/Pacific Islander, and 9.5% of Latina births are preterm.[1]

Perinatal depressive and anxiety symptoms are common and consequential, and also disproportionately affect low-income women and women of color. In a survey of California women who had given birth in the past year, a staggering 20.7% and 18.2% of women with incomes below the federal poverty level (FPL) reported experiencing elevated depressive symptoms during pregnancy and the postpartum period, respectively, compared to 7% and 9.2% of women above 200% FPL.[24] Prenatal depressive symptoms are associated with increased risk of PTB.[25–27] Moreover, perinatal depression is associated with psychological consequences for children, such as internalizing (e.g., depression) and externalizing (e.g., attention deficit hyperactivity disorder) disorders,[28] as well as higher health care expenditures[29] and infant health care utilization.[30] Although perinatal mental health research has largely focused on depression, elevated anxiety symptom severity is also highly prevalent[31,32]and associated with adverse perinatal outcomes.[33,34] These findings underscore the importance of effective and timely prevention and treatment of perinatal depression and anxiety.

Low-income women face many barriers to receiving high quality prenatal care that can impede full engagement in care. These barriers, particularly among women of color,[35–37] include feeling disrespected or discriminated against because of income, race, ethnicity, or language.[38–42] Exposures to chronic stressors, including racism, are known to be associated with an increased risk of PTB;[36,43,44] experiencing poor treatment in care can exacerbate these issues, and can lead to reduced engagement in prenatal care. In a systematic review of prenatal care experiences, low-income women were more likely to report stereotyping, long waits, rushed visits, and a perception that providers did not value them and that their care was mechanistic.[45] These experiences correlated with inconsistent access to and utilization of care.[46] Additionally, low-income women of color are more likely to report perceptions of racism and mistrust of health care providers than higher income women of color.[47] Nationally, 18-21% of Latina and Black women report experiencing poor treatment during prenatal care due to their race, culture, and/or primary language.[48] Low-income Black and Latina women in Fresno, Oakland, and San Francisco corroborate these findings, describing experiences with disrespectful care as the receipt of blatant and subtle discriminatory acts from staff and providers during prenatal care and labor and delivery.[39] There is a strong and persistent association between racial discrimination and initiation, engagement, and dissatisfaction with prenatal care,[35,36,39,41,42] emphasizing the need to improve women's prenatal care experiences. Finally, increased use of telehealth during the COVID-19 pandemic may exacerbate racial/ethnic and socioeconomic disparities in prenatal care, given barriers to telehealth use among low-income populations.[49] More nuanced understanding of women's prenatal care experiences is needed to inform comprehensive, coordinated models of care and support that address psychosocial and structural barriers for low-income women.[45]

The demographics of the Fresno County population (990,000 residents)[50] and its preterm birth risk profile is similar to other large urban settings with substantial economic and racial diversity. Fresno has the highest preterm birth rates in California: In 2015, it was 10.1% versus 8.5% in the state overall.[51] Like elsewhere in the US, Fresno's PTB disparities reflect longstanding societal inequities, with low-income and women of color bearing the greatest burden. Rates of poverty in Fresno County are high: 58.7% of women who recently delivered there had incomes at or below the FPL, and 72% relied on Medi-Cal (versus 40% and 50%, respectively, for all California women).[52,53] Moreover, in Fresno County, 15.7% of Black women, 13.1% of American Indian/Alaskan Natives, 10.1% of Asian/Pacific Islanders and 9.4% of Latinas (who constitute 62% of women who give birth in Fresno) experience a PTB compared to 9.1% of White women.[53]

There are key gaps in the available evidence regarding enhanced prenatal care programs that address social determinants of health for low-income families. The Comprehensive Prenatal Services Program (CPSP) was developed as a pilot project for 7,000 low-income women in 13 California counties;[54] it was later expanded to include all Medi-Cal recipients. In 1995, a statewide evaluation found that CPSP participants were 30% less likely to have a low-birth-weight infant compared to Medi-Cal recipients who did not use CPSP.[55] Although these findings were promising, selection bias and retrospective data limit their generalizability. There is a call to action by patient advocacy organizations to evaluate birth outcomes for women who utilize CPSP using rigorous data collection and fidelity measures.[56] Additionally, the coordination of additional social services for CPSP patients has not been evaluated. Comparing the effectiveness of this program component between CPSP/IC and Glow/GC will address systemic barriers low-income women face when accessing publicly funded programs.

Group prenatal care is endorsed by the American College of Obstetricians and Gynecologists[57] and the World Health Organization as a model that may improve birth outcomes.[58] However, evidence of its effectiveness in reducing preterm birth is mixed, leading these organizations to call for additional research. Meta-analyses found that while group prenatal care is viewed positively by women and is not associated with adverse health outcomes, there is insufficient evidence to conclude that it reduces preterm birth.[59] Even less evidence is available regarding the effectiveness of group prenatal care among low-income women, women of color or in women considered high risk as a result of maternal health.[59,60] In one study, Latinas who received group prenatal care attended visits more consistently than women who received individual care; however there was no difference in the rates of PTB or low birth weight newborns.[61] The authors recommended that randomized trials should be conducted with low-resourced, minority populations to address self-selection bias and improve validity. Although women using Glow/GC have reported high levels of satisfaction, evidence regarding whether this approach can result in lower PTB rates, improved mental health, or more respectful care as compared to CPSP/IC is not available.

In sum, the EMBRACE study is designed to fill critical gaps in evidence regarding the effectiveness of two enhanced prenatal care models in low-income, racially/ethnically diverse populations.

1.1.1 Conceptual framework

The modified socioecological framework[62] outlines the four interacting levels of personal and environmental factors that impact maternal and child health. The structural level refers to systems that evoke socioeconomic/cultural policies and norms; the community level pertains to care settings and resources that impact women's access; the interpersonal level is personal relationships; and the individual level is a woman's physical and mental health. Based on the framework and relevant literature, <u>we hypothesize that, compared to CPSP/IC, Glow/GC has greater capacity to improve mental health (primary aim), result in more positive care experience (secondary aim) and decrease PTB rates (exploratory aim) by incorporating social determinants of health, peer-to-peer processes, and process-of-care factors (see Figure 1).</u>

Figure 1. Conceptual Framework

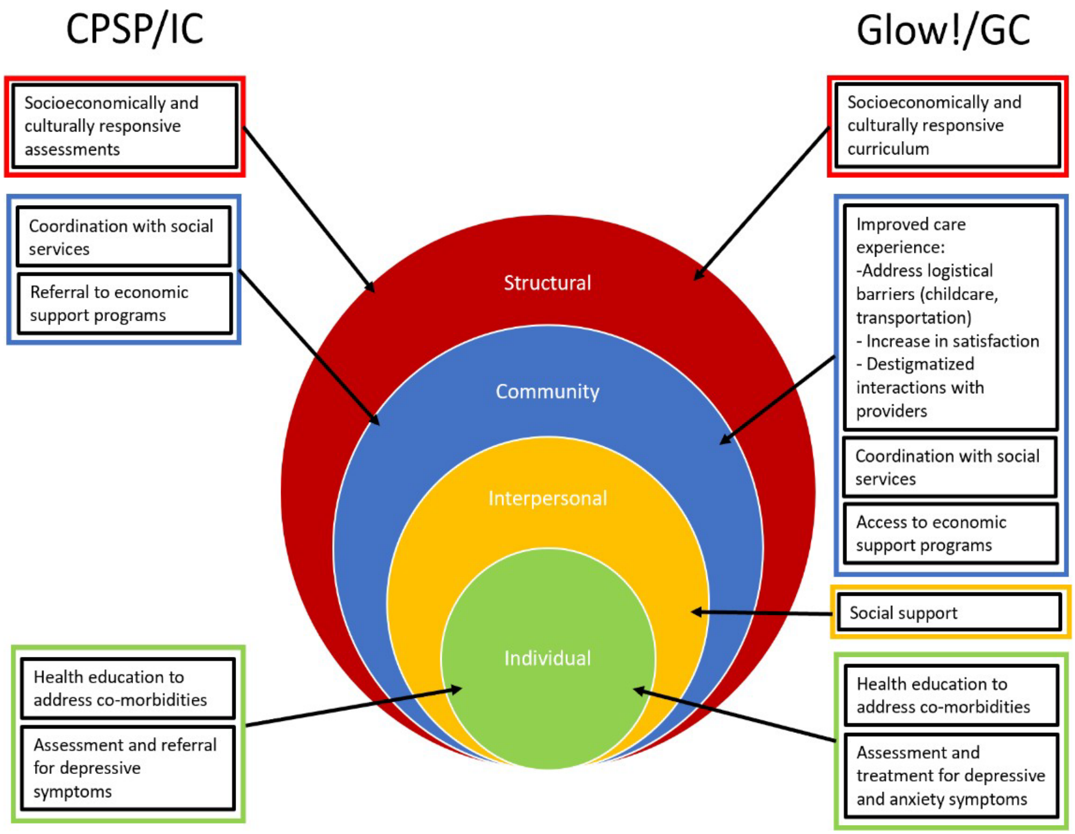

2.0 STUDY HYPOTHESES AND DESIGN

This randomized comparative effectiveness trial has three aims and each has primary and secondary hypotheses.

2.1 Hypotheses

The first aim of the study is to compare the effect of *Glow group prenatal care with wrap around services (Glow/GC) versus individual prenatal care with supplemental services covered by the California Department of Public Health Comprehensive Perinatal Services program (CPSP/IC)* on changes in depressive symptom severity (1° outcome) and anxiety symptom severity (2° outcome) from baseline (6-24 weeks gestation) to postpartum (3 months), using the Patient Health Questionnaire (PHQ-9)[63] and the Generalized Anxiety Disorder (GAD-7)[64] scale. The hypotheses for this study aim are:

Primary Hypothesis (Aim 1)

- Compared to women allocated to CPSP/IC, women allocated to Glow/GC will have greater reductions in baseline depressive symptom severity at 3 months postpartum.

Secondary Hypotheses (Aim 1)

Compared to women allocated to CPSP/IC, women allocated to Glow/GC will have:

- Greater reductions in baseline depressive symptom severity in the 3rd trimester.

- Greater reductions in baseline anxiety symptom severity at 3 months postpartum and in the 3rd trimester.

The second aim is to compare the effect of Glow/GC and CPSP/IC on participant-reported person-centered care, measured with Person-Centered Prenatal Care scale (PCPC)[66] and Person-Centered Maternity Care scale[67], respectful care measured with Mothers on Respect index (MORi)[35], and satisfaction with care, measured with Prenatal Care Satisfaction (PCS) scale.[65]

Primary Hypothesis (Aim 2)

- Compared to women allocated to CPSP/IC, women allocated to Glow/GC will report more person-centered care in the 3rd trimester.

Secondary Hypotheses (Aim 2)

Compared to women allocated to CPSP/IC, women allocated to Glow/GC will report:

- More person-centered care at 3 months postpartum.
- More respectful care in the 3rd trimester.
- More satisfaction with care in the 3rd trimester and at 3 months postpartum.

The third aim is to compare the effect of Glow/GC versus CPSP/IC on preterm birth rates and gestational age at delivery among low-income women. This is an exploratory aim.

Exploratory Primary Hypothesis (Aim 3)

- Compared to women allocated to CPSP/IC, women allocated to Glow/GC will have lower rates of preterm birth.

Exploratory Secondary Hypotheses (Aim 3)

- Compared to women allocated to CPSP/IC, women allocated to Glow/GC will give birth at higher gestational ages.

2.2 Study Design

This is a randomized comparative effectiveness trial to determine the effect of two enhanced prenatal care models, Glow/GC and CPSP/IC, on depressive and anxiety symptom severity; person-centered care, perceptions of respectful care, and satisfaction with prenatal care; and PTB rates and gestational age at delivery among 657 birthing people. Providers will be randomized to the order in which they offer the two models, and participants will be allocated to each of the comparators based on their provider and expected due date. Providers may select the mode of comparator delivery—either in person, via telehealth or a combination of both—according to personal preferences, health center regulations, and local public health guidelines. A CONSORT diagram of the study is presented as Figure 2.

2.3 Randomization

Both comparators will be offered within each site. At each site, participating providers will be randomly assigned to one of two recruitment tracks: recruiting initially for Glow/GC or recruiting initially for CPSP/IC. The two tracks will alternate between recruitment for Glow/GC and recruitment for CPSP/IC based on prespecified six-week ranges of estimated delivery dates (EDD), for the duration of the study. Based on the provider's randomization track and the participant's due date, participants will be placed in either the Glow/GC or CPSP/IC arm (see Figure 3 for an example of the treatment assignment for Provider A, who was randomly assigned to recruit for Glow/GC for the initial 6-week period of the study). This design optimizes the number of Glow/GC sessions offered by each practice (no more than 4 concurrent groups per clinician) and is ideal for clinic flow. Because the maximum number of women

who can be enrolled in a Glow cohort is 12, after 12 have enrolled, subsequent eligible women will be allocated to CPSP.

Figure 2. Consort diagram

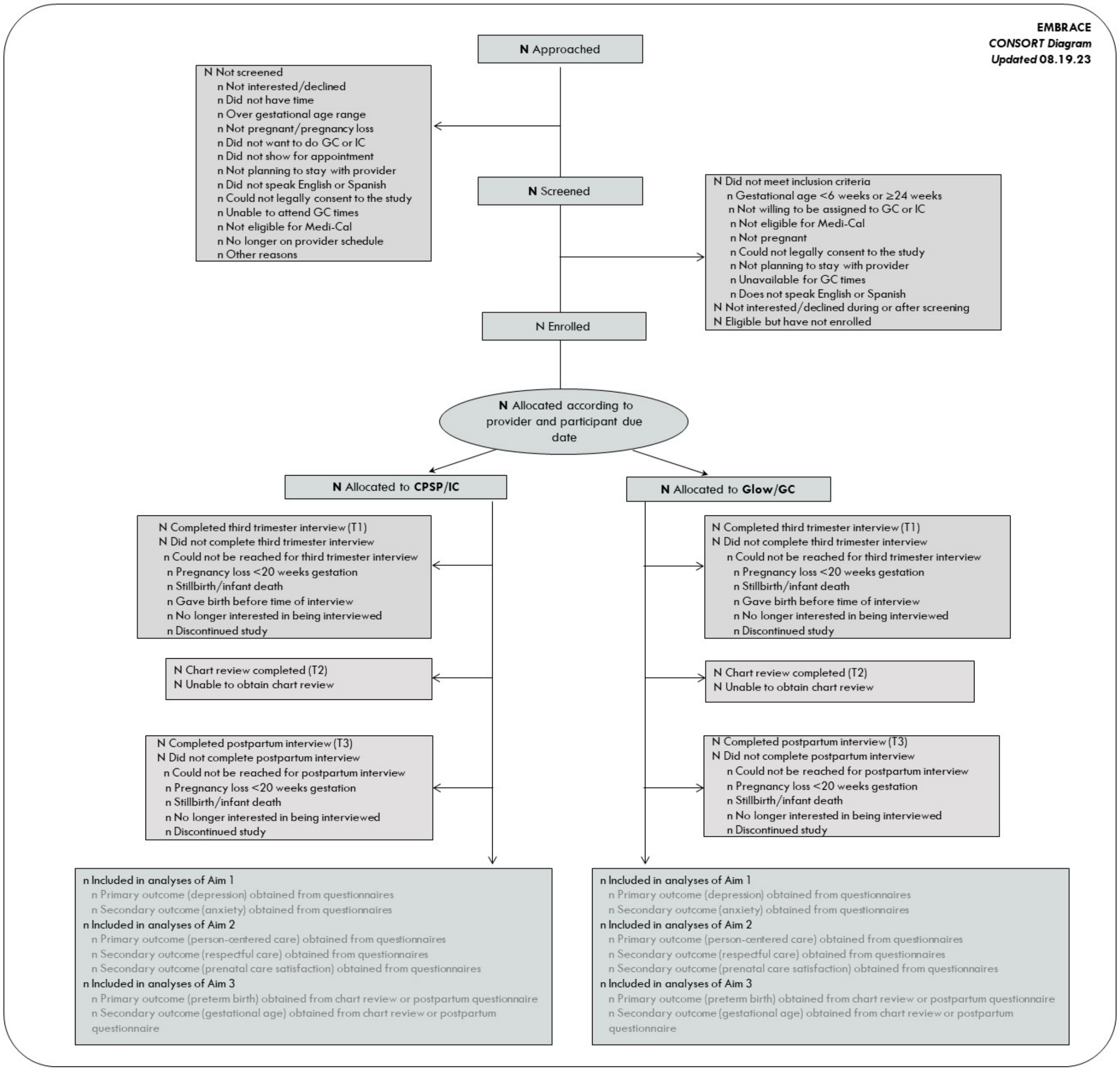

Randomization will be stratified by recruitment site and provider. If the site has more than one provider and can accommodate only one Glow group at a time, the first provider will be randomized to a starting comparator track and the second provider will start with the other comparator sequence. Randomization tables were generated in blocks of size 4 with Stata v16.0 using the runiform function, which utilizes the 64-bit Mersenne-Twister random number generator. RRSs will not assign participants to the Glow/GC or CPSP/IC arm until after completion of their baseline survey, at which point the RRS will inform the participant of her group assignment and enter it into the study database. The appropriate comparator assignment is determined utilizing a tool that does not reveal comparator

estimated due date ranges. To help ensure that the assessors of the primary and secondary outcomes are masked with respect to group assignment, a different staff member will conduct the third trimester and postpartum interviews and perform the medical chart abstraction.

Figure 3. Example Provider Recruitment Track with Randomization

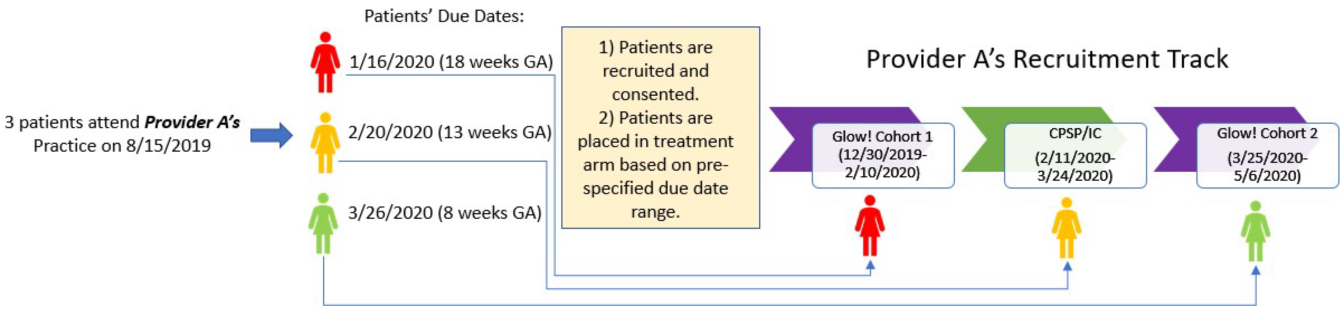

3.0 STUDY POPULATION

A total of 657 pregnant women who are between 8 weeks (or less than 8 weeks with pregnancy confirmed by ultrasound) and 24 weeks gestation and eligible for Medi-Cal will be enrolled into the study from one of the participating prenatal care provider sites in Fresno County. Fresno County has the highest rate of preterm birth in California and a high rate of poverty among pregnant women.[5,52] Provider sites in Fresno County are staffed by obstetrician-gynecologists, family medicine physicians, physician assistants, nurse-midwives, nurse practitioners, nurses, and other health care providers. Together, these sites serve a diverse patient population, both racially/ethnically and linguistically, and primarily consist of Medi-Cal recipients, similar to low-income care settings across the US.

Providers will be randomized to offer Glow/GC or IC/CPSP in 6-week intervals, and participants will be assigned to the intervention based on their due dates. Participants will be selected for the study according to the criteria in Sections 3.1 and 3.2. They will be approached, screened, and enrolled as described in Sections 5.1.1., 5.1.2., and 5.1.3. Issues related to participant retention and withdrawal from the study are described in Sections 3.3 and 3.4, respectively.

3.1 Inclusion Criteria

Women who meet all of the following criteria are eligible for inclusion in the study:

- ≤24 weeks, 6 days' gestation (with pregnancy confirmed by ultrasound if < 8 weeks) at enrollment
- Eligible for Medi-Cal (at or below 213% of the federal poverty level)
- Speak English or Spanish
- Willing to be assigned to Glow/GC or CPSP/IC to receive prenatal care

3.2 Exclusion Criteria

Women who meet any of the following criteria will be excluded from this study:

- Not planning to continue prenatal care with site provider
- Cannot legally consent to participate in the study
- Unable to speak the language of the sessions that are meeting
- Unable to attend group prenatal care session at the scheduled time

3.3 Participant Retention

Once a participant enrolls in EMBRACE, every effort will be made to retain them in the study for the entire follow-up period to minimize possible bias associated with loss to follow-up. Components of such procedures may include:

- Thorough explanation of the study interview schedule and procedural requirements during the informed consent process.
- Collection of email, phone, and address contact information during enrollment.
- Collection of an alternative contact's email and phone information after the consent process.
- Repeated contact attempts to schedule interviews.
- Immediate follow-up for missed interviews.
- Email and physical mail reminders.
- Retention gift sent by mail prior to the final interview.
- In-person approach at participant's prenatal care site if the participant cannot be reached through email, phone, or physical mail.

Contact attempts: RRSs will attempt to contact the participant up to 5 times and will record all contact attempts in the REDCap[68] database. After the fifth attempt, the RRS will reach out to the alternative contact. If attempting to reach the alternative contact does not result in contact with the participant, the RRS will attempt to get updated contact information from clinic staff. If the previous methods are all unsuccessful, the RRS will report the participant as lost to follow-up in the REDCap database on the "discontinuation, withdrawal, and loss to follow-up" form.

3.4 Participant Withdrawal

Participants who withdraw from their assigned intervention will be encouraged to complete all study questionnaires. Cards and a retention gift will be sent to promote study engagement. However, regardless of the participant retention methods used, participants may voluntarily withdraw from the study for any reason at any time. Participants also may be withdrawn if the study sponsor, regulatory authorities, or site IRB terminate the study prior to its planned end date.

The study team will evaluate participants withdrawn from the study on a case-by-case basis to determine whether birth outcomes may be collected from the chart and retained in analyses. A final determination will be made according to a participant's stated preference for use of her medical record data and the data use guidelines set forth in the informed consent form.

4.0 STUDY ARMS

4.1 Glow/GC (enhanced group prenatal care, eGPC)

Glow/GC, Comparator 1 for this study, is an enhanced group prenatal care program administered by First 5 Fresno County that offers co-located social services provided by established community agencies with independent funding streams targeting low-income families. The Glow/GC model pairs a trained staff facilitator with a licensed prenatal care provider from a practice site to provide billable prenatal care to the practice's own patients. Over the course of 8-11 sessions, 6-12 women (within a 6-week gestational age range) receive prenatal and postpartum medical care, risk assessments, social support, and gain knowledge and skills related to pregnancy, birth, and parenting. Glow/GC modifies the CenteringPregnancy®[69] curriculum, which incorporates ACOG recommendations for prenatal

care,[57] to include modules focusing on depression, anxiety, sleep, mindfulness, and how discrimination may affect birth outcomes. Spanish-language sessions and materials are available.

Additional Glow program components include childcare support with developmental assessments (in-person model) or in-home early learning activities for children ages 0 to 5 (telehealth model); education, referral, and treatment for perinatal mental health issues and conditions administered by the Department of Behavioral Health, Perinatal Wellness Program (both models); transportation stipends (both models); take-home food (in-person model) or food delivery (telehealth model) through local food banks and other community agencies; and direct coordination/enrollment with education, housing, economic assistance, and other local services as needed (both models). Glow/GC staff maintain a county-based, low-income resource guide and work continuously to build relationships and facilitate warm handoffs.

The current protocol incorporates the needs identified by participants, community members, and stakeholders who shared their feedback regarding telehealth modifications to meet required physical distancing and other challenges presented by the COVID-19 pandemic. The core components of Glow! sessions were retained and modified to fit the abilities of participants and providers to use telehealth. First 5 Fresno County manages the Glow/GC program and closely documents any changes to their program protocol and implementation. The EMBRACE project manager and project specialist will work closely with the agency to track these changes and communicate them to the rest of the EMBRACE team.

4.2 CPSP/IC (enhanced individual prenatal care, eIPC)

CPSP/IC is comparator 2 for this study and is administered by the Fresno County Department of Public Health, with oversight provided by the California Department of Public Health. Participants allocated to CPSP/IC will be invited to participate in CPSP assessments with a Comprehensive Perinatal Health Worker (CPHW) at their prenatal care site, where individual prenatal care will be provided. The individual clinical care provided to CPSP/IC participants will not differ from the care that is routinely provided by the site. The CPSP orientation (90-minute health assessment and education) and up to 3 additional assessments (45-minute health education sessions during the 2nd and 3rd trimesters and postpartum) will be conducted at the provider’s office or via telehealth, depending on the provider, patient, and clinic. CPSP participation is optional; services provided are Medi-Cal billable. CPHWs are trained to deliver client-centered, culturally sensitive care and develop an Individualized Care Plan with participants connecting them with services to help meet client-driven goals.

5.0 STUDY PROCEDURES

5.1 Overview

The RRS will perform a preliminary eligibility screening by reviewing site appointment logs or working with clinic staff to identify women who have completed or are scheduled for an initial obstetric appointment at a participating site. Women who are ≤24 weeks and 6 days’ gestation will be identified and approached for in-person or remote screening (according to physical distancing restrictions and clinic preferences). Women without appointments who are seeking prenatal care (walk-ins) will also be approached in person or by phone. When in person, the RRS will approach potentially eligible women in the site waiting room while the woman is waiting to see her provider, provide a brief explanation of the study, and ask if the woman is willing to answer a few questions to

determine if she is eligible for the study. If the woman agrees, the recruiter will administer the study screening questionnaire. If a woman is eligible for the study, the recruiter will provide her with a study summary sheet (e.g., detailed description of the study) including the risks, benefits and alternatives to participating. If the woman agrees to participate, the recruiter will review the study consent with her and answer any questions before asking the woman to sign the consent and medical record release and providing her with a copy of the study consent. For women who plan to give birth at Community Medical Centers (CMC), the consent process will include an additional hospital-specific consent and HIPAA release. Women who would like more time to consider whether to participate will be asked if the recruiter can contact her in a few days to see if she has any additional questions or, if preferred, the woman will be given the study phone number. Alternatively, the recruiter may ask to follow up with the woman at her next prenatal appointment to answer any questions and determine if she's reached a decision.

If time permits and the pregnant individual is interested in participating in the study, screening, consent, baseline questionnaire administration and comparator allocation will take place on the same day and immediately after screening is completed. If a participant is unable to complete the baseline questionnaire at the time of screening, the RRS will arrange a time to administer the baseline questionnaire and complete study enrollment.

Given potential physical distancing and other policies which may be in place at the time of enrollment, potential participant names and contact information may be collected over the phone from clinic staff, and recruitment may be conducted remotely. Signed consents will be obtained using DocuSign. DocuSign will automatically provide the participant with a copy of the consent. Alternatively, if the individual is unable or unwilling to complete the consent remotely, the option for in-person consent within Fresno County will be provided.

All study instruments will be administered in the language preferred by the participant, English or Spanish. After the participant completes the baseline questionnaire (see section 5.1.1) assignment to Glow/GC or CPSP/IC will take place based on provider randomization sequence and participant's due date. Participants allocated to Glow/GC will receive information on Glow/GC session times, location, and administrative contacts. A woman allocated to CPSP/IC will be contacted by a CPSP provider during subsequent prenatal care appointments.

Participants will be administered a third trimester (≥30 weeks' gestation) questionnaire by a RRS masked to the comparator group of the participant (see section 5.1.4. of the EMBRACE protocol). The ideal timing for the questionnaire will between 30-34 weeks' gestation. RRSs will attempt to conduct third trimester questionnaires by telephone but will administer questionnaires in person as needed.

After delivery, a medical record review will be performed by a study staff member, masked to intervention assignment, to ascertain the primary outcome of preterm birth and the secondary outcome of gestational age at delivery from post-randomization participants (see section 5.1.5.). The masked staff will also review the medical record to collect other clinical data from the delivery encounter including the secondary maternal and neonatal morbidity outcomes.

Participant-reported primary and secondary outcomes will be assessed with a three-month postpartum questionnaire (ideally at 10-14 weeks postpartum) (see section 5.1.6.). RRSs will attempt to conduct postpartum questionnaires by telephone but will administer questionnaires in person as needed.

Participants will receive $30 in remuneration for participating in the baseline, $50 in remuneration for participating in the third trimester questionnaire, and $50 for participating in the postpartum questionnaire.

5.1.1. Prenatal Care Medical Record Screening

Staff at participating clinics will provide site appointment logs or will work with the RRS to identify women who have completed or are scheduled for an initial prenatal appointment at a participating site and who are ≤24 weeks and 6 days' gestation.

5.1.2. Screening and Approach

Women identified as potentially eligible by clinic staff or through medical record screening will be approached in the clinic waiting area before the prenatal appointment or will be contacted remotely. Women without appointments who are seeking prenatal care (walk-ins) also will be approached at each provider site. Screening for eligibility will be administered by the RRS and all screening data will be entered in the REDCap database. (See Appendix 1 and 2 for screening questions). Pregnant persons will be confirmed eligible if they meet the eligibility criteria as previously specified (see section 3.1 and 3.2).

If the woman meets all eligibility criteria (screening takes approximately 10 minutes) and has enough time, informed consent and the baseline questionnaire will be administered on the same day (approximately 45 minutes). Otherwise, she will be contacted by the RRS to schedule a time to complete the enrollment process (obtain informed consent and complete the baseline questionnaire and enrollment) in conjunction with an upcoming prenatal care visit during her eligibility window. If the RRS is unable to find a prenatal care visit within the eligibility window, she will conduct the enrollment and baseline questionnaire at the prenatal care site, over the telephone, at the Central Valley Health Policy Institute or at another mutually agreed upon location.

A record of every woman approached will be kept in a REDCap database. Records of all women screened, regardless of eligibility, will be entered in the REDCap database before completing the baseline interviews.

5.1.3. Enrollment and Baseline Questionnaire (≤24 weeks and 6 days' gestation)

<u>Consent</u>. After the screening, signed informed consent and permission for release of medical records will be obtained from eligible participants using the study consent and HIPAA research authorization forms. Signed consents and HIPAAs will be obtained in person or remotely through DocuSign. Participants will complete the study consent and HIPAA forms for the delivery hospital and the prenatal care site. If the participant plans on giving birth at a Community Medical Center (CMC) facility, they will be asked to sign a CMC-specific HIPAA research authorization form and consent that has been approved by the CMC IRB (Appendices21; see sections 8.2 and 8.3 for more information).

<u>Baseline Questionnaire</u>. After the participant signs the study consent form, the RRS will administer the baseline questionnaire (Appendices 9 and 10). This questionnaire includes baseline measures of primary and secondary outcomes for depression, anxiety, discrimination and includes questions on demographics, COVID-19, health history, pregnancy history, stress, sleep, employment, income, housing, financial stress, food security, environmental exposures, loneliness and social support. If this process occurs in person, a print copy of the questionnaire will be offered to the participant to follow along as questions are read aloud. The RRS will keep track of the time at the start and end of the interview and note if there is an interruption to the interview lasting more than 5 minutes. The RRS will also record if anyone is with the participant during the interview. All questionnaire data will be collected and stored in the REDCap database.

Allocation. The comparator will be assigned according to the provider randomization table generated by the statistician co-investigator and by using the Comparator Assignment Tool created by the CVHPI team and managed by the project manager. At sites where there is more than one participating provider, it is possible that patients may receive care from multiple providers. At these locations, the RRS will ask participants which provider they consider to be their primary prenatal care provider. If they do not know, the RRS will list their provider in REDCap as the provider with whom they have an appointment on the day they are approached. If the participant identifies a specific participating provider, the RRS will list the specified provider, regardless of whom their appointment is with on the day they are approached. If assigned to Glow/GC, the RRS will provide the participant with the Glow/GC schedule and information needed to attend each session. A participant assigned to Glow/GC will have her information shared with the Glow/GC program coordinator and the assigned group care staff facilitator will follow up with her. This information is maintained in a binder located at each clinic and managed by the Glow/GC program coordinator and clinic staff. A participant assigned to CPSP/IC will have her information shared with a CPSP provider or clinic staff member, and the CPSP provider or clinic staff member will follow up with her.

Remuneration and follow-up reminder. Women who complete the baseline questionnaire will receive $30 in remuneration, which can be paid in person with cash or remotely through five different remote payment methods (CashApp, Venmo, PayPal, Zelle, and ApplePay). Participants will sign for receipt of remuneration, or will text confirmation for remote payment, and staff will reconcile records monthly. RRSs will send participants reminders for their follow-up interviews using the participant's preferred contact method in advance of the interview date.

5.1.4 Third Trimester Follow-up Interview *(≥30 weeks' gestation)*

The third trimester questionnaire will be completed with participants in both arms of the study. The goal of this interview is to check-in with the participant and collect data on the participant-reported secondary outcomes and covariates.

Third trimester questionnaire. The RRS will contact the participant to schedule a time to complete the questionnaire by telephone, at CVHPI, or at an upcoming prenatal care appointment, according to participant preference. The ideal timing for the interview will between 30-34 weeks' gestation and a minimum of 6 weeks after the baseline interview. The questionnaire will be administered by a different RRS than the one who administered the baseline questionnaire to ensure the RRS remains masked to the participant's allocation assignment. A copy of the third trimester questionnaire (Appendices 11 and 12) will be offered to the participant so she may follow along as questions are read aloud. The RRS will keep track of the time at the start and end of the interview and note if there is an interruption to the interview lasting more than 5 minutes.

During the interview, the participant will be asked where they plan to deliver. If the facility is different from the facility reported at baseline, the RRS will ask the participant to sign a HIPAA research authorization form for the new facility if necessary, and if required, a consent to release birth hospitalization medical records to the study team.

If the participant has experienced a stillbirth or infant death at ≥20 weeks gestation, the RRS will ask if they would like to complete a final exit interview with modified questions and their information will be recorded in the REDCap database (see Appendix 17 and 18). We will review the participant's medical record to abstract the clinical data. If participants experience a pregnancy loss prior to 20 weeks, the RRS will let them know they are no longer eligible for follow-up interviews and their information will be recorded in the REDCap database; their medical record data will not be abstracted or included in the study analyses. If the participant has already given birth at the time

of the third trimester questionnaire, they will not complete the interview and the postpartum interview will be completed.

Remuneration. Women who complete the third trimester questionnaire will receive $50. Participants will sign for receipt of remuneration and staff will reconcile records monthly. As noted above in section 5.1.3, payments can be made remotely.

5.1.5. Medical Record Abstraction (after delivery)

Participants' birth hospitalization medical record will be reviewed after delivery to abstract clinical data (Appendices 13). Abstraction of delivery outcome data will be performed by a UCSF team member who is masked to the comparator allocation. The preterm birth outcome will be derived from gestational age at delivery. We will collect information on other infant and maternal outcomes available in the delivery record.

5.1.6. Postpartum Questionnaire (10-14 weeks after delivery)

The three months postpartum questionnaire will be completed with participants in both arms of the study. The goal of this interview is to complete a final assessment of participants in the study and congratulate women on completing the study and the birth of their baby.

If the participant has lost the pregnancy or lost her child since the last interview, the RRS will contact her to complete a pregnancy loss or infant death form. The RRS will also report the pregnancy loss or infant death in the REDCap database on the "Discontinuation, withdrawal, and loss to follow-up form."

Postpartum Questionnaire. The RRS will contact participants 10 weeks after estimated or confirmed delivery to schedule a time for a telephone interview. If preferred by the participant, the interview will occur in person at the participant's home, CVHPI, or a public location of the participant's choosing. If the interview is conducted in person, a copy of the postpartum questionnaire (Appendices 14 and 15 will be offered to the participant via email or mail to follow along as questions are read aloud. The RRS will keep track of the time at the start and end of the interview and note if there is an interruption to the interview lasting more than 5 minutes. The RRS will also record if anyone is with the participant during the interview. Questionnaire data will be collected and stored in a REDCap database. After the questionnaire is completed, the RRS will mark the participant follow-up as complete in the final "Scheduling and follow-up" form (Appendix 22)

Remuneration. Women who complete the telephone or in-person interview will receive $50 in cash. Participants will sign for receipt of remuneration and staff will reconcile records monthly. As noted above in section 5.1.3, payments can be made remotely.

5.2 Study Timeline

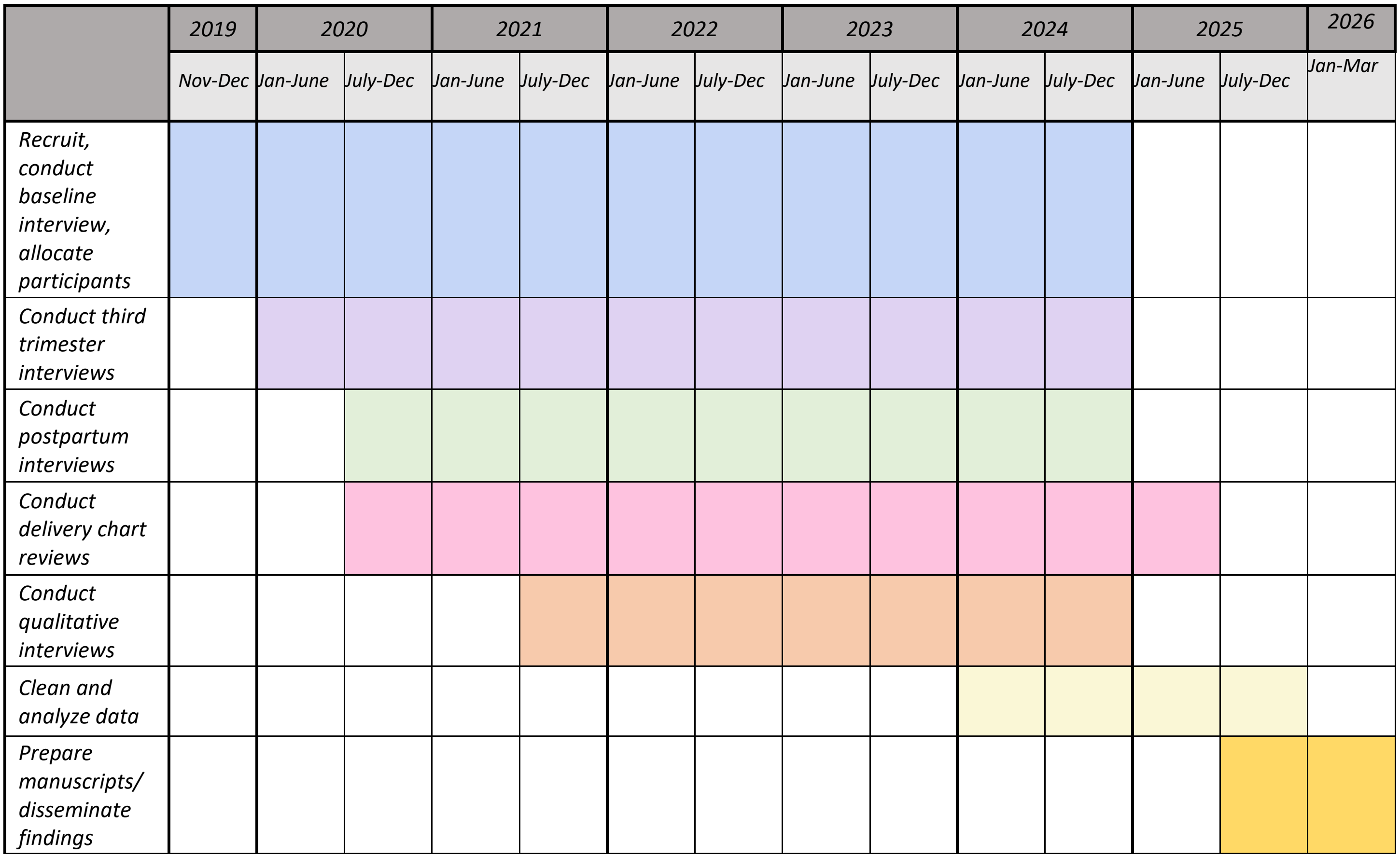

6.0 SAFETY MONITORING AND ADVERSE EVENT REPORTING

6.1 Safety Monitoring and Clinical Data Review

A multi-tiered safety review process will be followed for the duration of this study. As the study is minimal risk, the primary monitoring will occur by the PI and the institutional review boards. Close cooperation between the principal investigator, co-investigators, site director, project manager, RRSs, data manager, study statistician, and other study team members will be necessary to monitor participant safety and to respond to concerns in a timely manner. The investigative team will have monthly conference calls during the period of study implementation and additional ad hoc calls will be convened as needed.

The site director and the principal investigator will be responsible for ensuring monitoring of potential adverse effects related to study participation from participants or participating sites. The research staff will have clear directions for whom to contact regarding any issues that may arise during the research study. RRSs will report all adverse events to the site director within 72 hours of event occurrence. The site director will then report to the PI. These issues will also be discussed during monthly investigator calls.

All participants will have a prenatal care provider with whom they can consult regarding issues that arise during their prenatal care.

The site director and project manager are responsible for continuous close monitoring and management of adverse events (AE) in accordance with the protocol for AE reporting at their home institution.

Types of adverse events (AES)

- Internal (on-site) adverse events
- External (off-site) adverse events
- Expected adverse event
- Unexpected adverse event
- Serious adverse event (SAE)
- Hold on study accrual or other study activity

All adverse events, whether directly related or unrelated to study participation will be documented and kept in study files. The site director is responsible for the initial evaluation and reporting of safety information and for alerting the investigative team if unexpected concerns arise.

6.2 Reporting Requirements for this Study

The investigator team will assess AE causality in terms of overall study participation and make an independent determination as to whether the AE was thought to be related to any study-related activity. We will use the following definitions to assess the AE relationship to study participation:

- **Definitely related**

  An AE is definitely related to study participation if it is clear that the event was caused by study participation. A definitely related event has a strong temporal relationship and an alternative cause is unlikely.

- **Probably related**

  An AE is probably related when there is a reasonable possibility that the event is likely to have been caused by study participation. The AE has a timely relationship to the study procedure(s) and follows a known pattern of response, but a potential alternative cause may be present.

- **Possibly related**

  An AE is possibly related when there is a reasonable possibility that the event might have been caused by study participation. A possibly related event may follow no known pattern of response and an alternative cause seems more likely. In other circumstances, there may be significant uncertainty about the cause of the event, or a possible relationship to study participation cannot reasonably be ruled out.

- **Unrelated**

  The cause of the AE is known and the event is in no way related to any aspect of study participation. If there is any uncertainty regarding AE causality then the event must be assessed as possibly related to research participation and reported to the IRB as indicated. Often, the cause of an unrelated AE is disease progression.

The site director will report an adverse event to the study PI who will report to the local IRB if study staff determines it may qualify as an Unanticipated Problem or Adverse Event because the event meets all three criteria listed below:

- Unanticipated in severity or frequency AND

- At least *possibly* related to the study intervention AND
- Is Serious OR not serious but suggests placing subjects or others at greater risk

All AEs will be reported to the study team within 72 hours of recognition by study staff.

6.3 Interim analysis and data oversight

As risks associated with study participation are minimal, no plans are made for interim analysis of quantitative data. Data integrity and confidentiality will be continually monitored by the study data manager and the principal investigator by creating monthly reports of recruitment and weekly periodic checks on data quality, including examination of frequency of values for variables to look for outliers.

7.0 STATISTICAL CONSIDERATIONS

7.1. Endpoints

7.1.1 Primary Endpoint Aim 1

Consistent with the primary Aim 1 hypothesis, that women who receive Glow/GC will report lower depression symptom severity than women who receive CPSP/IC, the following endpoint will be assessed from: (1) baseline to third trimester, (2) third trimester to postpartum and (3) from baseline to postpartum.

- Changes in depressive symptom severity assessed using the Patient Health Questionnaire (PHQ-9) developed by Kroenke et al. (2001)[63].

7.1.2. Secondary Endpoint Aim 1

Consistent with the secondary Aim 1 hypothesis, that women who receive Glow/GC will report lower anxiety symptom severity than women who receive CPSP/IC, the following endpoint will be assessed from: (1) baseline to third trimester, (2) third trimester to postpartum and (3) from baseline to postpartum.

- Changes in anxiety symptom severity, assessed using the 7-item Generalized Anxiety Disorder (GAD-7) scale developed by Spitzer et al. (2006)[64].

7.1.3. Primary Endpoint Aim 2

Consistent with the primary Aim 2 hypothesis, that women who receive Glow/GC will report more person-centered prenatal care than women who receive CPSP/IC, the following endpoint will be assessed at third trimester and postpartum.

- Extent to which the participant feels that the prenatal care they received was person-centered, assessed using the Person-Centered Prenatal Care (PCPC) [66] scale developed by Afulani et al. (2021).

7.1.4. Secondary Endpoint Aim 2

Consistent with the secondary Aim 2 hypothesis, that women who receive Glow/GC will report more person-centered maternity care and more respectful and higher satisfaction with prenatal care than women who receive CPSP/IC, the following endpoints will be assessed at postpartum and third trimester.

- Extent to which the participant feels that the labor and delivery care they received was person-centered, assessed using the Person-Centered Maternity Care-US (PCMC-US) [67] scale developed by Afulani et al. (2021).
- Extent to which the participant feels that the prenatal care they received was respectful, assessed using the Prenatal Care Satisfaction (PCS) Scale developed by Raube et al. (1998)[65].
- Extent to which the participant feels satisfied with the prenatal care they received, assessed using the Mothers on Respect Index (MORi) developed by Vedam et al. (2017)[35].

7.1.5. Primary Endpoint Aim 3

Consistent with the primary Aim 3 hypothesis, that women who receive Glow/GC will be less likely than women who receive CPSP/IC to have a preterm birth, the following endpoint will be assessed from the delivery medical record review.

- Whether the participant had a baby born less than 37 weeks gestation, as noted in the participant's medical record.

7.1.6. Secondary Endpoint Aim 3

Consistent with the secondary Aim 3 hypothesis, that women who receive Glow/GC will have babies born at higher gestational ages than women who receive CPSP/IC, the following endpoint will be assessed from the delivery medical record review.

- The gestational age at which the baby was born (number of weeks), as noted in the participant's medical record.

7.2 Sample Size

We conducted sample size calculations for change in depressive symptom severity. We powered our study to detect meaningful changes in depressive and anxiety symptoms. Using an alpha of .05 and power of .85, we will be able to detect a standardized effect size of 0.25 with a sample size of 578 individuals in total (289 per arm). We are assuming a retention rate of 88% (as per our previous study and experience in the current study) and power of 85% (per PCORI's request). A standardized effect size of 0.2 to 0.3 is widely considered to be a small effect size.[107] We therefore plan to enroll 657 participants in the study, and, with a retention rate of 88%, we will have > 85% power to detect small effect sizes for these outcomes. Adjustment for covariates in multivariable analyses for numeric outcomes may make the residual SD smaller than the raw SD, improving precision and power.

7.3 Inclusion of Women and Minorities

As this study compares the effect of two models of enhanced prenatal care, we are, by necessity, restricting participant enrollment to women of reproductive age. We have selected 10 clinical sites which serve a racially/ethnically diverse patient population, many of whom have Medi-Cal coverage, to ensure that our findings are relevant to low-income women and women of color who are disproportionately affected by preterm birth, perinatal depressive and anxiety symptoms, and disrespectful prenatal care in the US.

7.4 Masking

The study participant and the RRS who administers the screening and baseline interview will be aware of the participant's comparator assignment. Third trimester interviews, postpartum

interviews, and delivery medical record review will be conducted by a different staff person to ensure the assessor of the primary and secondary outcomes is masked to the participant's comparator assignment. There is one exception to this plan: if there is only one bilingual RRS on staff, they may need to complete third trimester and postpartum interviews with women they recruited and enrolled.

7.5 Data Analysis

Primary and secondary analyses will include all participants based on treatment assignment, according to intention-to-treat principles. We expect our experimental design with allocation of treatment sequence by provider to lead to minimal imbalance at the participant level. Nonetheless we will collect data and adjust for potential confounders. Confounding information will be collected via REDCap[68,71,72] questionnaires at baseline, 3rd trimester, and postpartum. Medical records will be linked by participant name and date of birth and abstracted after delivery to obtain data for our clinical outcomes (preterm birth and gestational age at delivery) and covariates. Maternal comorbidities will also be recorded from the medical records. Adherence will be quantified as the number of prenatal care visits in each treatment model, as opposed to initial assignment. To assess the effectiveness of different durations of participation in either treatment, we will conduct a secondary analysis limited to 1) participants who have attended at least one group or individual care session (after randomization) and 2) participants who have attended at least one group or individual care session (after randomization). For all analyses, we will first examine descriptive statistics, check modeling assumptions, and present effect estimates along with associated confidence intervals. Data analysis will be conducted while masked to treatment assignment.

Aim 1 Hypothesis: Women assigned to Glow/GC will have greater reduction in depressive **(1°)** and anxiety **(2°)** symptom severity at 3 months postpartum (1° time point) and in the 3rd trimester (2° time point) than women assigned to CPSP/IC.

Aim 1 Analyses: We will compare changes in depressive (PHQ-9) and anxiety (GAD-7) symptom severity from baseline to three months postpartum and 3rd trimester. The multivariate analyses will follow an analytic plan similar to that of Aim 1, using linear mixed models to assess primary and secondary outcomes. We will conduct separate analyses comparing symptom exacerbation from baseline to 3rd trimester and baseline to 3 months postpartum. **Exploratory analyses (1a):** Previous work among pregnant women found rates of elevated depressive symptoms were 19.9% and 17.1% for Black and Latina women, respectively, compared to 9.5% of White women.[24] We will explore the disparities in maternal mental health for Black and Latina women in our sample. Exploratory Aim 2a will follow the same analysis plan as 1a.

Aim 2 Hypothesis: Women assigned to Glow/GC will report more person-centered care **(1°)**, respectful care **(2°)**, and prenatal care satisfaction **(2°)** than women assigned to CPSP/IC, at 3 months postpartum (1° timepoint) and in the 3rd trimester (2° timepoint).

Aim 2 Analyses: We will use quantitative and qualitative methods to assess patient perceptions of person-centered care, respectful care, and satisfaction with care. The quantitative analysis will follow methods similar to those described in Aim 1, using linear mixed models for the PCPC and PCMC-US, MORi, and PCSS numeric outcomes. **Exploratory analyses (2a)** will follow the same plan above for the Black and Latina subgroups. **For Aim 2b,** in-depth interviews will focus on experiences of respectful and disrespectful care during the current pregnancy or in the past, experiences of racism in care or in other settings, and barriers/enablers of accessing care or services in their family/social network. The study will generate digital audio recordings and

transcripts encrypted when in transit. Data will be entered, coded, memoed, and organized using the Dedoose software program,[73] which allows for open coding, theoretical memoing, and detailed conceptual matrixing of transcript and memo data within and across participants. Sorting functions allow for cross-group comparison by code or characteristic and for establishing a system of inter-coder reliability. We will store all data on a secure server at UCSF, accessible only to the investigative team via password-protected, encrypted computers.

Aim 3 Hypotheses (exploratory): Women assigned to Glow/GC will have lower rates of preterm birth **(primary (1°))** and will give birth at higher gestational ages **(secondary (2°)** than women assigned to CPSP/IC.

Aim 3 Analyses: We will describe simple differences in proportions of preterm birth and mean gestational age by treatment group. We will examine models comparing treatment assignment to Glow/GC versus CPSP/IC including the covariates, using logistic mixed models (for preterm birth) and linear mixed models (for gestational age), with clustering by practice and provider. We will examine assumptions of normality for the gestational age outcome, and deviations from linearity for covariates. **Exploratory subanalyses (3a):** We will explore preterm birth rates and gestational age at delivery among Black and Latina participants in each treatment arm, to examine whether Glow/GC and CPSP/IC may be more or less effective in these subgroups. These aims will be exploratory in nature, as we do not anticipate having a sufficient sample to have high power to test our hypotheses in these groups. We will assess effectiveness within subgroups based on race/ethnicity and language and report the p-value for interaction.

We will begin to code data immediately after the first phase of interviews are completed, in bimonthly data meetings that will include interview staff and co-investigators. We will take extensive notes during meetings to document emergent concepts and themes for codebook development. Codes will be developed mirroring the topics outlined in interview guides, which we will develop prior to study initiation. Regular data analysis meetings will create a constructive dialogue that both triangulate the quantitative and qualitative data collected in Aim 2. While reviewing qualitative data, we will note reoccurring themes (open coding). While completing open coding, we will write theoretical and methodological memos consistent with qualitative analysis methodologies.[74–81] We will finalize a codebook and code and enter all data into Dedoose. We will review selected transcripts to check for inter-coder reliability. Staff will maintain the coding database and produce outputs for analysis and generate data summaries.

*Missing outcomes and covariates***:** As baseline data will be collected at time of treatment assignment, and clinical outcomes will be obtained from medical record review, we do not estimate substantial missing data for Aim 3. We plan to reduce missing data for Aims 1 and 2 through our retention plan. We will record all reasons for study withdrawal and missing data. Participants lost-to-follow up will undergo medical record review for the outcomes of preterm birth and gestational age. Our mixed model analyses offer robust protection against data that are missing at random.[82] We will assess missing data across study covariates and time points and use multiple imputation if it is appreciable. Pregnancies that end in miscarriage, abortion or fetal death prior to 20 weeks gestation will not be included in analysis.

*Heterogeneity of treatment effect (HTE)***:** We will assess if the effectiveness of Glow/GC compared with CPSP/IC differs by practice or patient characteristics through interaction analyses. Pre-specified analyses of treatment heterogeneity include: complications of pregnancy, parity, race/ethnicity, provider type (CNM, MD, NP). We will also conduct exploratory analyses on additional covariates (social support, site, size of practice or location, prior experiences of racism) to assess treatment differences. HTE will be assessed using interactions between

treatment assignment and each proposed effect modifier tested with a cut-point of ($p<.10$) for HTE.

Avoidance of bias: The use of randomization of treatment initiation within practices allows us to minimize the risks of confounding and selection bias. More efficient designs with less susceptibility to bias are unfortunately not feasible in this study, as the treatment is administered at the practice level and needs to be incorporated without substantial interruption to clinical processes. To enhance generalizability of the study, we are recruiting from a diverse low-income patient population including English- and Spanish-speakers, with study staff and all assessment tools available in both languages. While we cannot mask treatment assignment to providers, participants or study staff, we will make every effort to ensure equivalent study procedures across both groups. Chart review and data analysis will be performed by study staff masked to treatment assignment to reduce ascertainment bias.

8.0 HUMAN SUBJECTS CONSIDERATIONS

8.1 Ethical Review

All study procedures, consent forms, study questionnaires and recruitment materials were reviewed and approved by the CSU Fresno and UCSF Institutional Review Boards (IRB). Any subsequent modifications to study procedures or documents after initial approval will be submitted for review and approval. Review will be with respect to scientific content and compliance with applicable research and human subjects regulations.

IRB review of the study protocol will occur at least annually. Study staff at CSU Fresno and UCSF will provide safety and progress reports to the IRBs at least annually and within three months of study termination or completion. These reports will include the total number of participants enrolled in the study, the number of participants who completed the study, all changes in the research activity, and all unanticipated problems involving risks to human subjects or others.

8.2 Informed Consent

Written informed consent will be obtained from each study participant prior to enrollment using a consent form approved by the local IRB in accordance with all applicable regulations. A copy of the study informed consent form will be offered to the participant at enrollment (Appendices 3 and 4).

8.3 HIPAA

Permission for release of medical records for ascertainment of clinical endpoints will be obtained from participants with the Health Insurance Portability and Accountability (HIPAA) research authorization form, which documents the personal health information that may be released, to whom it may be released, how it may be used, when permission for its release expires, and under what terms permission may be cancelled (Appendices 5 and 6).

8.4 Risks

This is a minimal risk research study commensurate with what might be expected in standard prenatal care. However, some interview questions are sensitive in nature and participants may feel uncomfortable answering. For example, the PHQ-9 scale includes measures of severity of depressive symptoms.

Risk will be minimized as follows. First, all participants will be informed, prior to participation, that they are free to terminate their participation at any time or to decline to answer any questions or participate in any part of the study. During the consent process, we will ensure that the participant understands this principle and feels free to exercise this option at any time. Second, study

questionnaires will be administered by RRSs in a private area and participants will be advised that they are free to skip any question they are uncomfortable answering. Finally, the RRS will enter participant responses directly in REDCap which will be programmed to alert the RRS to scores > 0 on item 9 of PHQ-9 (i.e., endorses "thoughts that you would be better off dead, or thoughts of hurting yourself in some way") and prompt the RRS to follow the study protocol.

If a study participant scores > 0 on item 9 of PHQ-9, the recruiter will ask the participant if she has received any counseling or help regarding those feelings. If the participant responds affirmatively, the recruiter will encourage her to contact this person. The RRS will refer the participant to Perinatal Wellness Center and urge her to call the Central Valley Suicide Prevention Hotline. Additionally, all women with scores > 0 on item 9 of PHQ-9 will be asked to consider contacting their prenatal care provider. Periodic screening for depression is now standard practice in prenatal care.

If RRSs have questions or concerns, they can contact Dr. Jennifer Felder, PhD, a study co-investigator and licensed psychologist with expertise in perinatal mental health, at 805-708-9056 to review the situation.

8.5 Benefits

There are no direct benefits to study participants. However, some participants enjoy contributing to scientific knowledge about prenatal care. Additionally, women assigned to Glow/IC may enjoy talking with other pregnant women while attending group prenatal care sessions.

The study will help low-income pregnant people and their providers determine which approach to enhanced prenatal care is most effective in reducing the risk of preterm birth, lowering symptoms of depression and anxiety, and providing care that feels satisfying and respectful. Furthermore, if shown to be effective, the study will generate evidence for policy makers and payers to expand access to enhanced group prenatal care for low-income women.

8.6 Incentives

Participants will be compensated for their time and effort in this study through remuneration of $30 for the baseline questionnaire and $50 for the third trimester and postpartum questionnaires. Participants will also be sent reminder cards for questionnaires and a baby beanie with a card when their child is born.

8.7 Confidentiality

Confidentiality will be maintained by administering study questionnaires in a private area and asking participants how they wish to be communicated with. Participant study information will not be released without the written permission of the participant except as necessary for monitoring by the NIH and/or the site IRB.

9.0 ADMINISTRATIVE PROCEDURES

9.1 Study Coordination

Study implementation will be directed by this study protocol. Protocols for training and study team communication are covered in sections 9.1.1. and 9.1.2. Section 9.1.3 reviews database management and data quality monitoring plans. Use of information is described in Section 9.2.

9.1.1 Trainings

The site director, project manager, and the RRC at CVHPI will train all recruitment team members. Topics covered may include:

- Research ethics and confidentiality
- CITI and HIPAA trainings
- Study background, justification and aims
- Inclusion and exclusion criteria, eligibility screening
- In-person and remote interview scheduling
- Approaching and contacting potentially eligible participants
- Etiquette for in-person approach in the clinic waiting area
- Etiquette for remote approaches by phone
- Documenting approaches
- Informed consent (remote and in-person)
- Comparator assignment protocol
- Questionnaire administration (in-person and remote)
- Baseline questionnaire
- Third trimester questionnaire
- Postpartum questionnaire
- Qualitative interview training
- Equipment
- Tablets
- Laptops
- Zoom scheduling and recording
- Remuneration
- Tracking of cash payments
- Tracking of remote payments
- Retention
- Withdrawal and discontinuation
- Health literacy and the social determinants of health
- Diversity and inclusion

The data manager at UCSF will train all project staff on topics related to database entry with REDCap and will monitor data quality. This may include:

- Forms for following study participants and scheduling subsequent interviews
- Approach, screening, contact, and consent forms
- Baseline, third trimester, and postpartum forms
- Stillbirth or Infant death forms
- Entering data for participants who are lost to follow up, had pregnancy losses prior to 20 weeks, and participant withdrawals or discontinuations
- Using weekly data quality reports to monitor enrollment and completion of study questionnaires
- Creating reports and data visualizations for study staff

9.1.2 Study Communication

Calls and in-person meetings will be scheduled throughout the study period and on an as-needed basis to ensure clear communication, collegial collaboration, and rapid response to any challenges that may arise.

<u>Investigator Calls</u>

The PI will lead monthly conference calls with study staff. Topics to be discussed may include:

- Project timelines
- Site recruitment plans
- Enrollment and retention reports
- Study design and implementation
- Questionnaire revisions (as necessary)
- Data analysis plans, progress and results
- Manuscript and abstract plans and progress
- Review of draft manuscripts and abstracts
- IRB renewals and modifications
- Budgets and contracts
- Other study management issues as needed

<u>Research Coordinator Meetings</u>

The RRC at CVHPI will hold daily meetings with the RRSs, and the project manager, at a minimum, will attend weekly with the site director participating as needed. Any new project manager should participate daily in these calls for at least one month. Coordinator meetings will cover topics including:

- Current totals for approached, scheduled for enrollment, enrolled, scheduled for interviews, followed-up, consents completed, and status uploading to REDCap, by each site
- Successes, challenges, and new ideas for:
    - Screening and approaching patients
    - Reaching recruitment targets
    - Utilizing technology (tablets, printers, Wi-Fi)
    - Administering questionnaires and qualitative interviews
    - Scheduling and coordinating follow-up interviews
    - Any issues needing attention at the medical provider level
    - Other issues as needed

9.2 Database Management and Data Quality Monitoring

Comparator assignment, survey responses, and medical record abstraction data for all sites will be collected and stored in REDCap databases hosted centrally at UCSF. Data will be maintained behind an institutional firewall and accessible through a web portal via login credentials known only to qualified study staff. Comparator assignment will be masked to RRSs during data collection with the except of the one bilingual RRS on staff. RRSs can maintain their own tracking and recruitment logs for each site as long as the final forms are entered into the local REDCap database. Protected health information, such as patient names, medical record number, estimated due date, date of birth, telephone number, mailing address, and email address will be de-identified after enrollment to all other study staff. A unique study identifier will be generated for each participant to link records within the database.

Data from REDCap will be used to develop monthly recruitment and enrollment reports. Study data will be transferred to statistical software for cleaning, reporting and analysis. Data will be exported from REDCap to perform data visualizations for study team meetings.

9.3 Use of Information and Publications

A description of this clinical trial will be available at http://www.ClinicalTrials.gov. Presentation and publication of the results of this study will be governed by guidelines determined by the study team and, as necessary and appropriate, by their associated institutions' policies. Any presentation, abstract, or manuscript will be approved by the study PI prior to submission.

10.0 IMPORTANT CHANGES TO ORIGINAL PROTOCOL

Version of protocol	Date	Description of change	Brief rationale
1	1/31/20	Original protocol	n/a
2	7/9/20	Adapt eGPC comparator for telehealth	During the early days of the COVID-19 pandemic, before the COVID vaccine had been developed and tested in pregnant individuals, pregnant people could not gather in close proximity, precluding in-person group prenatal care.
3	8/23/23	Elevate Aim 2 (mental health) to make it the primary aim and make Aim 1 (preterm birth) an exploratory aim. While the content of the three aims remained unchanged, their order was revised midway through the study. This resulted in a diminution in the necessary sample size from 2600 to 657. This change was approved by PCORI and all participating IRBs and was registered on ClinicalTrials.gov. The study period also was extended through 3/31/26.	The decision was made in response to recruitment challenges in the wake of the COVID-19 pandemic, as well as emerging external evidence indicating that group prenatal care did not significantly reduce preterm birth rates compared to individual prenatal care but was associated with meaningful reductions in depressive symptoms. The study period was extended to enable us to recruit the required sample size.

11.0 REFERENCES

1. Martin JA, Hamilton BE, Osterman MJK, Driscoll AK, Drake P. Births: Final data for 2016. *National Vital Statistics Reports*. 2018;67(1).

2. Mathews TJ, Macdorman MF, Thoma ME. Infant mortality statistics from the 2013 period linked birth/infant death data set. *National Vital Statistics Reports*. 2015;64(9):1-30.

3. Saigal S, Doyle LW. An overview of mortality and sequelae of preterm birth from infancy to adulthood. *The Lancet*. 2008;371(9608). doi:10.1016/S0140-6736(08)60136-1

4. Romero R, Dey SK, Fisher SJ. Preterm labor: One syndrome, many causes. *Science*. 2014;345(6198):760-765. doi:10.1126/science.1251816

5. Rosenthal L, Earnshaw VA, Lewis TT, et al. Changes in experiences with discrimination across pregnancy and postpartum: Age differences and consequences for mental health. *American Journal of Public Health*. 2015;105(4):686-693. doi:10.2105/AJPH.2014.301906

6. Rosenthal L, Lobel M. Explaining racial disparities in adverse birth outcomes: Unique sources of stress for Black American women. *Social Science and Medicine*. 2011;72(6). doi:10.1016/j.socscimed.2011.01.013

7. Central Valley Health Policy Institute. Determinants of Infant Mortality in Fresno County: California State University, Fresno. Published online 2015. Accessed August 10, 2021. http://www.fresnostate.edu/chhs/cvhpi/documents/AAIM%201-7-2016%20Final.pdf

8. Ickovics JR, Kershaw TS, Westdahl C, et al. Group prenatal care and perinatal outcomes: A randomized controlled trial. *Obstetrics and Gynecology*. 2007;110(2 I). doi:10.1097/01.AOG.0000275284.24298.23

9. Ickovics JR, Reed E, Magriples U, Westdahl C, Rising SS, Kershaw TS. Effects of group prenatal care on psychosocial risk in pregnancy: Results from a randomised controlled trial. *Psychology and Health*. 2011;26(2):235-250. doi:10.1080/08870446.2011.531577

10. Westdahl C, Milan S, Magriples U, Kershaw TS, Rising SS, Ickovics JR. Social support and social conflict as predictors of prenatal depression. *Obstetrics and Gynecology*. 2007;110(1):134-140. doi:10.1097/01.AOG.0000265352.61822.1b

11. Chae SY, Chae MH, Kandula S, Winter RO. Promoting improved social support and quality of life with the CenteringPregnancy® group model of prenatal care. *Archives of Women's Mental Health*. 2017;20(1):209-220. doi:10.1007/s00737-016-0698-1

12. Heberlein EC, Picklesimer AH, Billings DL, Covington-Kolb S, Farber N, Frongillo EA. The comparative effects of group prenatal care on psychosocial outcomes. *Archives of Women's Mental Health*. 2016;19(2):259-269. doi:10.1007/s00737-015-0564-6

13. Martin JA, Hamilton BE, D P, et al. National Vital Statistics Reports Births: Final Data for 2013. *Statistics*. 2015;64(1).

14. Jacob J, Kamitsuka M, Clark RH, Kelleher AS, Spitzer AR. Etiologies of NICU deaths. *Pediatrics*. 2015;135(1). doi:10.1542/peds.2014-2967

15. Basten M, Jaekel J, Johnson S, Gilmore C, Wolke D. Preterm birth and adult wealth: Mathematics skills count. *Psychological Science*. 2015;26(10):1608-1619. doi:10.1177/0956797615596230

16. Ely DM, Driscoll AK. Infant mortality in the United States, 2018: Data from the period linked birth/infant death file. *National Vital Statistics Reports*. 2020;69(7).

17. Boivin A, Luo ZC, Audibert F, et al. Risk for preterm and very preterm delivery in women who were born preterm. *Obstetrics and Gynecology*. 2015;125(5):1177-118. doi:10.1097/AOG.0000000000000813

18. Boardman JP. Preterm Birth: Causes, Consequences and Prevention. *Journal of Obstetrics and Gynaecology*. 2008;28(5). doi:10.1080/01443610802243047

19. Jelliffe-Pawlowski LL, Baer RJ, Blumenfeld YJ, et al. Maternal characteristics and mid-pregnancy serum biomarkers as risk factors for subtypes of preterm birth. *BJOG: An International Journal of Obstetrics and Gynaecology*. 2015;122(11):1484-1493. doi:10.1111/1471-0528.13495

20. Blumenshine P, Egerter S, Barclay CJ, Cubbin C, Braveman PA. Socioeconomic disparities in adverse birth outcomes: A systematic review. *American Journal of Preventive Medicine*. 2010;39(3). doi:10.1016/j.amepre.2010.05.012

21. Culhane JF, Goldenberg RL. Racial disparities in preterm birth. *Seminars in Perinatology*. 2011;35(4):234-239. doi:10.1053/j.semperi.2011.02.020

22. Lu MC, Halfon N. Racial and ethnic disparities in birth outcomes: a life-course perspective. *Maternal and child health journal*. 2003;7(1):13-30. doi:10.1023/A:1022537516969

23. Markus AR, Krohe S, Garro N, Gerstein M, Pellegrini C. Examining the association between Medicaid coverage and preterm births using 2010–2013 National Vital Statistics Birth Data. *Journal of Children and Poverty*. 2017;23(1):79-94. doi:10.1080/10796126.2016.1254601

24. Health CDoP. Symptoms of depression during and after pregnancy. 2018. Accessed Sept. 3, 2018.

25. Felder JN, Epel E, Lewis JB, et al. Depressive symptoms and gestational length among pregnant adolescents: Cluster randomized control trial of CenteringPregnancy® plus group prenatal care. *Journal of Consulting and Clinical Psychology*. 2017;85(6): 574-584. doi:10.1037/ccp0000191

26. Jarde A, Morais M, Kingston D, et al. Neonatal outcomes in women with untreated antenatal depression compared with women without depression: A systematic review and meta-analysis. *JAMA Psychiatry*. 2016;73(8):826-837. doi:10.1001/jamapsychiatry.2016.0934

27. Stein A, Pearson RM, Goodman SH, et al. Effects of perinatal mental disorders on the fetus and child. *The Lancet*. 2014;384(9956):1800-1819. doi:10.1016/S0140-6736(14)61277-0

28. Goodman SH, Rouse MH, Connell AM, Broth MR, Hall CM, Heyward D. Maternal depression and child psychopathology: A meta-analytic review. *Clinical Child and Family Psychology Review*. 2011;14(1):1-27. doi:10.1007/s10567-010-0080-1

29. Dagher RK, McGovern PM, Dowd BE, Gjerdingen DK. Postpartum depression and health services expenditures among employed women. *Journal of Occupational and Environmental Medicine*. 2012;54(2):210-215. doi:10.1097/JOM.0b013e31823fdf85

30. Roubinov DS, Felder JN, Vieten C, et al. Maternal depressive symptoms and infant healthcare utilization: The moderating role of prenatal mindfulness. *General Hospital Psychiatry*. 2018;53:82-83. doi:10.1016/j.genhosppsych.2018.01.001

31. Britton JR. Maternal anxiety: Course and antecedents during the early postpartum period. *Depression and Anxiety*. 2008;25(9):793-800. doi:10.1002/da.20325

32. Fairbrother N, Janssen P, Antony MM, Tucker E, Young AH. Perinatal anxiety disorder prevalence and incidence. *Journal of Affective Disorders*. 2016;200:148-155. doi:10.1016/j.jad.2015.12.082

33. Ding XX, Wu Y le, Xu SJ, et al. Maternal anxiety during pregnancy and adverse birth outcomes: A systematic review and meta-analysis of prospective cohort studies. *Journal of Affective Disorders*. 2014;159:103-110. doi:10.1016/j.jad.2014.02.027

34. Dunkel Schetter C, Tanner L. Anxiety, depression and stress in pregnancy: Implications for mothers, children, research, and practice. *Current Opinion in Psychiatry*. 2012;25(2):141-148. doi:10.1097/YCO.0b013e3283503680

35. Vedam S, Stoll K, Rubashkin N, et al. The Mothers on Respect (MOR) index: measuring quality, safety, and human rights in childbirth. *SSM - Population Health*. 2017;3:201-210. doi:10.1016/j.ssmph.2017.01.005

36. Rankin KM, David RJ, Collins JW. African American women's exposure to interpersonal racial discrimination in public settings and preterm birth: The effect of coping behaviors. *Ethnicity and Disease*. 2011;21(3):370-376.

37. Women UNCotSo. Convention on the elimination of all forms of discrimination against women. Published 2016. Accessed September 2, 2018. http://www.un.org/womenwatch/daw/cedaw/

38. Bowser D, Hill K. Exploring Evidence for Disrespect and Abuse in Facility-Based Childbirth Report of a Landscape Analysis. *Harvard School of Public Health University Research Co, LLC*. Published online 2010.

39. McLemore MR, Altman MR, Cooper N, Williams S, Rand L, Franck L. Health care experiences of pregnant, birthing and postnatal women of color at risk for preterm birth. *Social Science and Medicine*. 2018;201:127-135. doi:10.1016/j.socscimed.2018.02.013

40. Morton CH, Henley MM, Seacrist M, Roth LM. Bearing witness: United States and Canadian maternity support workers' observations of disrespectful care in childbirth. *Birth*. 2018;45(3):263-274. doi:10.1111/birt.12373

41. Ben J, Cormack D, Harris R, Paradies Y. Racism and health service utilisation: A systematic review and meta-analysis. *PLoS ONE*. 2017;12(12). doi:10.1371/journal.pone.0189900

42. Attanasio L, Kozhimannil KB. Patient-reported communication quality and perceived discrimination in maternity care. *Medical Care*. 2015;53(10). doi:10.1097/MLR.0000000000000411

43. Giurgescu C, Zenk SN, Dancy BL, Park CG, Dieber W, Block R. Relationships among neighborhood environment, racial discrimination, psychological distress, and preterm birth in African American

women. *JOGNN - Journal of Obstetric, Gynecologic, and Neonatal Nursing*. 2012;41(6). doi:10.1111/j.1552-6909.2012.01409.x

44. Braveman P, Heck K, Egerter S, et al. Worry about racial discrimination: A missing piece of the puzzle of Black-White disparities in preterm birth? *PLoS ONE*. 2017;12(10). doi:10.1371/journal.pone.0186151

45. Novick G. Women's experience of prenatal care: An integrative review. *Journal of Midwifery and Women's Health*. 2009;54(3):226-237. doi:10.1016/j.jmwh.2009.02.003

46. Lia-Hoagberg B, Rode P, Skovholt CJ, et al. Barriers and motivators to prenatal care among low-income women. *Social Science and Medicine*. 1990;30(4):487-495. doi:10.1016/0277-9536(90)90351-R

47. Benkert R, Peters RM, Clark R, Keves-Foster K. Effects of perceived racism, cultural mistrust and trust in providers on satisfaction with care. *Journal of the National Medical Association*. 2006;98(9):1532-1540.

48. Attanasio L, Kozhimannil KB. Health care engagement and follow-up after perceived discrimination in maternity care. *Medical Care*. 2017;55(9):830-833. doi:10.1097/MLR.0000000000000773

49. Ensuring the growth of telehealth during covid-19 does not exacerbate disparities in care. Health Affairs Blog. Published 2020. Accessed August 30, 2021. https://www.healthaffairs.org/do/10.1377/hblog20200505.591306/full/

50. Census US. Fresno County, California. Published 2017. Accessed September 2, 2018. https://www.census.gov/quickfacts/fact/table/fresnocountycalifornia/PST045217.

51. March of Dimes. Premature birth report card. Published online 2017. Accessed September 2, 2018. https://www.marchofdimes.org/peristats/tools/reportcard.aspx?frmodrc=1&reg=06

52. California Department of Public Health. Maternal and infant health assessment (MIHA) survey data snapshots. Published 2018. Accessed September 2, 2018. https://www.cdph.ca.gov/Programs/CFH/DMCAH/MIHA/Pages/Data-and-Reports.aspx

53. California Department of Public Health. Maternal and infant health assessment (MIHA) survey. County and regional data snapshots for subgroups, 2013-2015. Published online 2018. Accessed September 2, 2021. https://www.cdph.ca.gov/Programs/CFH/DMCAH-/MIHA/CDPH%20Document%20Library/20132015/SnapshotCo_Fresno_20132015_MaternalCharacteristics.pdf

54. California Department of Public Health. Comprehensive perinatal services program. Published 2018. Accessed September 2, 2018. https://www.cdph.ca.gov/-Programs/CFH/DMCAH/CDPH%20Document%20Library/Communications/Profile- CPSP.pdf

55. Lennie JA, Klun JR, Hausner T. Low-birth-weight rate reduced by the obstetrical access project. *Health Care Financing Review*. 1987;8(3).

56. Maternal and Child Health. Closing the gaps: Psychosocial services to improve maternal and child health.

57. ACOG Committee Opinion No. 731 Summary: Group Prenatal Care. *Obstetrics & Gynecology*. 2018;131(3):616-618. doi:10.1097/AOG.0000000000002526

58. World Health Organization. *WHO Recommendation on Group Antenatal Care.*; 2019.

59. Carter EB, Temming LA, Akin J, et al. Group prenatal care compared with traditional prenatal care: a systematic review and meta-analysis. *Obstetrics and Gynecology*. 2016;128(3):551-561. doi:10.1097/AOG.0000000000001560

60. Catling CJ, Medley N, Foureur M, et al. Group versus conventional antenatal care for women. *Cochrane Database of Systematic Reviews*. 2015(2):Cd007622. doi:10.1002/14651858.CD007622.pub3

61. Trudnak TE, Arboleda E, Kirby RS, Perrin K. Outcomes of Latina women in CenteringPregnancy group prenatal care compared with individual prenatal care. *Journal of Midwifery and Women's Health*. 2013;58(4):396-403. doi:10.1111/jmwh.12000

62. Bronfenbrenner U. *Ecological Systems Theory*. Vol 6. Kingsley Publishers; 1989.

63. Kroenke K, Spitzer RL, Williams JBW. The PHQ-9: Validity of a brief depression severity measure. *Journal of General Internal Medicine*. 2001;16(9):606-613. doi:10.1046/j.1525-1497.2001.016009606.x

64. Spitzer RL, Kroenke K, Williams JBW, Löwe B. A brief measure for assessing generalized anxiety disorder: The GAD-7. *Archives of Internal Medicine*. 2006;166(10):1092-1097. doi:10.1001/archinte.166.10.1092

65. Raube K, Handler A, Rosenberg D. Measuring satisfaction among low-income women: a prenatal care questionnaire. *Maternal and child health journal*. 1998;2(1):25-33. doi:10.1023/A:1021841508698

66. Afulani PA, Altman MR, Castillo E, et al. Development of the person-centered prenatal care scale for people of color. *American Journal of Obstetrics and Gynecology*. 2021. doi:10.1016/j.ajog.2021.04.216. Epub ahead of print. PMID: 33862014.

67. Afulani PA, Altman MR, Castillo E, et al. Adaptation of the Person-Centered Maternity Care scale for people of color in the United States. *In review.* 2021: doi:10.1101/2021.05.06.21256758

68. Research Electonic Capture (REDCap). Accessed August 30, 2021. https://redcap.ucsf.edu

69. Benediktsson I, McDonald SW, Vekved M, McNeil DA, Dolan SM, Tough SC. Comparing CenteringPregnancy® to standard prenatal care plus prenatal education. *BMC pregnancy and childbirth*. 2013;13 Suppl 1. doi:10.1186/1471-2393-13-s1-s5

70. Felder JN, Laraia B, Coleman-Phox K, et al. Poor sleep quality, psychological distress, and the buffering effect of mindfulness training during pregnancy. *Behavioral Sleep Medicine*. 2018;16(6):611-624. doi:10.1080/15402002.2016.1266488

71. Harris PA, Taylor R, Thielke R, Payne J, Gonzalez N, Conde JG. Research electronic data capture (REDCap)-A metadata-driven methodology and workflow process for providing translational research informatics support. *Journal of Biomedical Informatics*. 2009;42(2):377-381. doi:10.1016/j.jbi.2008.08.010

72. Harris PA, Taylor R, Minor BL, et al. The REDCap consortium: Building an international community of software platform partners. *Journal of Biomedical Informatics*. 2019;95:103208. doi:10.1016/j.jbi.2019.103208

73. Dedoose Version 8.0.35, web application for managing, analyzing, and presenting qualitative and mixed method research data. Published online 2018. Accessed August 30, 2021. www.dedoose.com.

74. Sandelowski MJ. Editorial: Justifying qualitative research. *Research in Nursing and Health*. 2008;31(3):193-195. doi:10.1002/nur.20272

75. Sandelowski M, Leeman J. Writing usable qualitative health research findings. *Qualitative Health Research*. 2012;22(10):1404-1410. doi:10.1177/1049732312450368

76. Ayakaka I, Ackerman S, Ggita JM, et al. Identifying barriers to and facilitators of tuberculosis contact investigation in Kampala, Uganda: A behavioral approach. *Implementation Science*. 2017;12(1):33. doi:10.1186/s13012-017-0561-4

77. Chaisson LH, Katamba A, Haguma P, et al. Theory-informed interventions to improve the quality of tuberculosis evaluation at Ugandan health centers: A quasi-experimental study. *PLoS ONE*. 2015;10(7):e0132573. doi:10.1371/journal.pone.0132573

78. Handley MA, Santos MG, McClelland J. Reports from the field: Engaging learners as interpreters for developing health messages — designing the 'Familias Sin Plomo' English as a Second Language curriculum project. *Global Health Promotion*. 2009;16(3):53-58. doi:10.1177/1757975909339773

79. Fontil V, Lyles CR, Schillinger D, et al. Safety-net institutions in the US grapple with new cholesterol treatment guidelines: A qualitative analysis from the phoenix network. *Risk Management and Healthcare Policy*. 2018;11:99-108. doi:10.2147/RMHP.S156311

80. Thompson LM, Diaz-Artiga A, Weinstein JR, Handley MA. Designing a behavioral intervention using the COM-B model and the theoretical domains framework to promote gas stove use in rural Guatemala: A formative research study. *BMC Public Health*. 2018;18(1):253. doi:10.1186/s12889-018-5138-x

81. Yeuen K, Situ M, Handley M, McClean I, Rundall T, Schillinger D. Ecology matters: safety net patients' perspectives of diabetes self-management support strategies- a study from the UCSF Collaborative Research Network. *Asian-Pacific Journal of General Practice*. Published online 2009;1(1):1-15.

82. Gibbons RD, Hedeker D, Dutoit S. Advances in analysis of longitudinal data. *Annual Review of Clinical Psychology*. 2010;6:79-107. doi:10.1146/annurev.clinpsy.032408.153550
