# Supplementary material for: Enhanced Prenatal Care Models and Postpartum Depression: The EMBRACE Randomized Clinical Trial
Source: JAMA Netw Open. 2026 Feb 16;9(2):e2559883. doi: 10.1001/jamanetworkopen.2025.59883 (PMC12910397; doi:10.1001/jamanetworkopen.2025.59883)
Supplement: Supplement 2. — eMethods. eResults. eTable 1. Unique and common features across prenatal care delivery models and enhancements. eTable 2. Imputed means (standard deviations), within-group effect sizes, and between-group effect sizes in the Latine and Black subgroups eTable 3. Imputed means (standard deviations), within-group effect sizes, and between-group effect sizes in the subgroups with elevated depression risk eTable 4. Imputed means (standard deviations), within-group effect sizes, and between-group effect sizes for exploratory stress and sleep outcomes (n=674) [file jamanetwopen-e2559883-s002.pdf]

Supplemental Online Content

Felder JN, Leon-Martinez D, Karasek D, et al. Enhanced prenatal care models and postpartum depression: the EMBRACE randomized clinical trial. *JAMA Netw Open*. 2026;9(2):e2559883. doi:10.1001/jamanetworkopen.2025.59883

eMethods.
eResults.
eTable 1. Unique and common features across prenatal care delivery models and enhancements.
eTable 2. Imputed means (standard deviations), within-group effect sizes, and between-group effect sizes in the Latine and Black subgroups
eTable 3. Imputed means (standard deviations), within-group effect sizes, and between-group effect sizes in the subgroups with elevated depression risk
eTable 4. Imputed means (standard deviations), within-group effect sizes, and between-group effect sizes for exploratory stress and sleep outcomes (n=674)

This supplemental material has been provided by the authors to give readers additional information about their work.

© 2026 Felder JN et al. *JAMA Network Open*.

eMethods

Changes to Trial Protocol

Initially preterm birth was the primary outcome of the trial. Midway through the study, we reordered the aims such that mental health became the primary aim with depression as the primary outcome and preterm birth was designated exploratory due to recruitment challenges in the wake of the COVID-19 outbreak and emerging external evidence that group prenatal care did not reduce preterm birth rates when compared to individual prenatal care, and that it was associated with significant reductions in depressive symptoms. This change was approved by PCORI, the UCSF and CSU Fresno IRBs, and registered on ClinicalTrials.gov prior to any data analysis. Other important changes to the trial after it commenced are described in the Protocol.

Participants

All participants were asked to self-report their race and ethnicities using categories defined by the investigators to enable us to conduct subgroup analyses among Black and Latine participants, both of whom are known to be at increased risk of depression and other adverse pregnancy and birth outcomes.

Adverse Events

See the Protocol for information about how adverse events were defined. The study did not include a systematic assessment of harms (e.g. via surveys or interviews). Adverse events were identified by spontaneous participant report. No formal adjudication process was used.

Interventions

During the COVID-19 pandemic, the eGPC model was adapted for virtual delivery, incorporating alternative strategies for providing wrap-around supports (e.g., grocery deliveries, at-home children's activities during sessions) and at-home tools for health assessments (e.g., home-based blood pressure monitoring). Sessions were conducted via Zoom. For the comparator, eIPC, providers adopted telehealth to varying degrees; however, some patients continued to receive in-person care, which operated under stricter policies regarding additional attendees, mask requirements, and social distancing.

Statistical Analysis

We also conducted sensitivity analyses among participants who attended at least 1 prenatal session (group or individual) after randomization and among those who attended at least 4 sessions after randomization. Heterogeneity of treatment effects were examined based on 3 pre-specified modifiers. First, we evaluated changes in depressive and anxiety symptom severity among participants who identified as Black or Latine. Second, we investigated whether GPC buffered against depressive symptom exacerbation in participants with a self-reported history of depression. Third, we examined whether eGPC reduced depressive symptoms among those with elevated symptom severity at baseline (i.e., PHQ-9 score ≥ 10). Finally, we explored whether changes in sleep disturbance (PROMIS) and perceived stress (PSS) varied by prenatal care type.

eResults

Adverse Events

No spontaneously reported adverse events met the criteria for IRB reporting.

Sensitivity Analyses

Results for the primary and secondary outcomes did not substantively differ in the subgroups of participants who attended at least 1 or at least 4 prenatal care sessions after randomization.

Heterogeneity of Treatment (HTE) Effects

Our first exploratory HTE analysis focused on the subgroups of participants who identified as Black (n=65) or Latine (n=515) and yielded no statistically significant between-group differences in changes in depressive and anxiety symptoms (eTable 2). Among Latine participants, both care types led to statistically significant reductions from baseline to postpartum in depressive and anxiety symptoms, with moderate and small standardized effect sizes, respectively. Among Black participants, both care types led to statistically significant reductions from baseline to postpartum in depressive, but not anxiety symptoms, with small-to-moderate standardized effect sizes.

Our second and third exploratory HTE analyses focused on two subgroups of participants at particularly high risk for depressive symptom exacerbation in the perinatal period: those with a self-reported history of depression (n=154), and those with elevated symptom severity at baseline (PHQ-9 ≥10; n=117). No statistically significant between-group differences in changes in depressive symptoms emerged in either subgroup (eTable 3). Among participants with a self-reported history of depression, both care types led to statistically significant reductions in depressive symptoms from baseline to postpartum, with small-to-moderate standardized effect sizes. Among participants with elevated symptom severity at baseline, both care types led to statistically significant reductions in depressive symptoms from baseline to postpartum, with large standardized effect sizes exceeding the threshold for a minimal clinically important difference of -2.0 on the PHQ-9 (i.e., -5.0 and -5.1 from baseline to

© 2026 Felder JN et al. *JAMA Network Open*.

third trimester for eGPC and eIPC, respectively; and -7.4 and -6.5 from baseline to postpartum for eGPC and eIPC, respectively).

Other A Priori Exploratory Analyses

Our final exploratory analysis examined whether changes in sleep disturbance and perceived stress varied by prenatal care type. Relative to participants assigned to individual prenatal care, participants assigned to GPC did not experience significantly greater reductions in perceived stress or sleep disturbance (eTable 4).

© 2026 Felder JN et al. *JAMA Network Open*.

eTable 1. Unique and common features across prenatal care delivery models and enhancements.

Feature	CenteringPregnancy (GPC)	Enhanced Group Prenatal Care (eGPC)	Traditional Prenatal Care (IPC)	Comprehensive Perinatal Services Program (CPSP/eIPC)
**Prenatal care**				
**Clinical care**	10 group sessions following ACOG visit schedule; 90–120 minutes	10–11 enhanced group sessions following ACOG visit schedule; 90–120 minutes	One-on-one prenatal care following ACOG visit schedule	One-on-one prenatal care following ACOG visit schedule
**Peer support**	Group discussion and shared experiences are central to the model	Group discussion and shared experiences are central to the model	None	None
**Features addressing mental health**				
**Perinatal mental health screening and referral**	Peer support; mental health needs typically addressed through external referral	Screening at every session, coordinated referral, and follow-up	Brief screening only (e.g., depression); referrals may vary by clinic	Structured psychosocial assessment, counseling, and coordinated referral and follow-up
**Additional mental health support**	None	Mindfulness included in every session; curriculum addenda on mental health topics (e.g., sleep, worry) and informational community presentations on mental health resources	None	None
**Enhancements addressing SDoH**				

© 2026 Felder JN et al. *JAMA Network Open*.

**Informational support for community resources and social services**	Not systematically included; referrals depend on risk factors	Integrated presentations from community agencies and structured resource-sharing (e.g., WIC, lactation support, perinatal mental health, paid family leave)	Not systematic; referrals vary by clinic	CPSP (re)assessments and referrals (e.g., psychosocial sterssors, social needs, substance abuse); resources offered as part of individualized care plan that can include referrals for services
**Wrap-around supports (childcare, transportation, food)**	Not included	Provided to address barriers to participation and support needs	Not included	Not included

© 2026 Felder JN et al. *JAMA Network Open*.

eTable 2. Imputed means (standard deviations), within-group effect sizes, and between-group effect sizes in the Latine and Black subgroups

	Mean (SD)			Within-Group Standardized Mean Differences[1] (95% CI)		Between-Group Standardized Mean Differences[2] (95% CI)	
	Baseline	Third Trimester	Postpartum	Baseline to Third Trimester	Baseline to Postpartum	Baseline to Third Trimester	Baseline to Postpartum
**Latine Subgroup (n=515)**							
PHQ-9							
eGPC	5.7 (4.9)	4.9 (4.2)	3.4 (4.1)	-0.1 (-0.2,0.0)	-0.5 (-0.7,-0.3)***	0.1 (-0.0,0.3)	0.1 (-0.2,0.3)
eIPC	4.4 (3.8)	3.8 (3.8)	2.8 (3.7)	-0.2 (-0.4,-0.1) ***	-0.6 (-0.7,-0.4)***		
GAD-7							
eGPC	4.1 (4.6)	3.9 (4.2)	3.1 (4.1)	0.0 (-0.1,0.2)	-0.2 (-0.3,-0.0)*	0.1 (-0.1,0.3)	0.0 (-0.2,0.3)
eIPC	3.1 (3.8)	3.1 (3.8)	2.5 (3.8)	-0.1 (-0.2,0.0)	-0.2 (-0.4,-0.1)**		
**Black Subgroup (n=65)**							
PHQ-9							
eGPC	6.2 (4.0)	6.2 (4.5)	4.9 (4.6)	-0.1 (-0.5,0.4)	-0.4 (-0.8,-0.0)*	0.1 (-0.4,0.6)	0.2 (-0.4,0.8)
eIPC	7.3 (4.2)	6.4 (4.1)	4.5 (4.5)	-0.2 (-0.5,0.1)	-0.6 (-0.9,-0.2)**		
GAD-7							
eGPC	4.6 (3.2)	4.8 (5.0)	5.2 (5.0)	-0.0 (-0.6,0.5)	0.1 (-0.3,0.4)	-0.1 (-0.8,0.5)	0.3 (-0.3,0.9)
eIPC	5.3 (4.2)	5.6 (3.9)	3.8 (4.5)	0.1 (-0.2,0.4)**	-0.3 (-0.6,0.1)		

Note. Negative effect sizes indicate a reduction in symptoms. * p<.05; **p<.01; ***p<.001; ***Abbreviations***. SD = Standard deviation; CI = Confidence interval; PHQ-9 = Patient Health Questionnaire-9; GAD-7= Generalized Anxiety Disorder Scale; eGPC = enhanced group prenatal care; eIPC = enhanced individual prenatal care. [1]Estimated standardized change in mean values over time within a group, adjusted for baseline value of outcome, self-reported history of a mental health condition, calendar time at enrollment (as a restricted cubic spline with four knots), and language of the questionnaire. [2]Standardized difference between groups in estimated change over time in mean values, adjusted for baseline value of outcome self-reported history of a mental health condition, calendar time at enrollment (as a restricted cubic spline with four knots), and language of the questionnaire

© 2026 Felder JN et al. *JAMA Network Open*.

eTable 3. Imputed means (standard deviations), within-group effect sizes, and between-group effect sizes in the subgroups with elevated depression risk

	Mean (SD)			Within-Group Standardized Mean Differences[1] (95% CI)		Between-Group Standardized Mean Differences[2] (95% CI)	
	Baseline	Third Trimester	Postpartum	Baseline to Third Trimester	Baseline to Postpartum	Baseline to Third Trimester	Baseline to Postpartum
**History of depression (n=154)**							
PHQ-9							
eGPC	8.2 (5.3)	6.8 (4.7)	5.7 (4.8)	-0.2 (-0.5,0.0)	-0.4 (-0.7,-0.2)**	0.1 (-0.4,0.5)	0.0 (-0.4,0.5)
eIPC	6.2 (4.1)	5.6 (4.3)	4.6 (5.3)	-0.3 (-0.5,-0.0)*	-0.5 (-0.7,-0.2)***		
**Elevated depression at baseline (n=114)**							
PHQ-9							
eGPC	13.4 (3.1)	8.2 (4.4)	5.8 (4.9)	-1.1 (-1.3,-0.8)***	-1.5 (-1.7,-1.3)***	0.0 (-0.4,0.4)	-0.2 (-0.5,0.2)
eIPC	12.2 (2.6)	7.4 (5.2)	6.0 (5.9)	-1.1 (-1.4,-0.8)***	-1.3 (-1.6,-1.0)***		

Note. Negative effect sizes indicate a reduction in symptoms. * p<.05; **p<.01; ***p<.001; ***Abbreviations***. SD = Standard deviation; CI = Confidence interval; PHQ-9 = Patient Health Questionnaire-9; GAD-7= Generalized Anxiety Disorder Scale; eGPC = enhanced group prenatal care; eIPC = enhanced individual prenatal care. [1]Estimated standardized change in mean values over time within a group, adjusted for baseline PHQ-9, self-reported history of a mental health condition, calendar time at enrollment, and language of the questionnaire. [2]Standardized difference between groups in estimated change over time in mean values, adjusted for baseline PHQ-9, self-reported history of a mental health condition, calendar time at enrollment, and language of the questionnaire.

© 2026 Felder JN et al. *JAMA Network Open*.

eTable 4. Imputed means (standard deviations), within-group effect sizes, and between-group effect sizes for exploratory stress and sleep outcomes (n=674)

	Mean (SD)			Within-Group Differences over Time[1] (95% CI)		Between-Group Differences[2] (95% CI)	
	Baseline	Third Trimester	Postpartum	Baseline to Third Trimester	Baseline to Postpartum	Baseline to Third Trimester	Baseline to Postpartum
PSS							
eGPC	13.7 (7.4)	12.6 (8.3)	11.6 (7.7)	-0.1 (-0.2,0.0)	-0.3 (-0.4,-0.1)***	-0.1 (-0.2,0.1)	-0.1 (-0.2,0.1)
eIPC	12.2 (7.3)	12.2 (8.9)	11.3 (7.5)	-0.0 (-0.2,0.1)	-0.2 (-0.3,-0.1)**		
PROMIS Sleep							
eGPC	50.3 (12.0)	52.8 (11.2)		0.3 (0.2,0.5)***		0.1 (-0.0,0.2)	
eIPC	47.8 (12.7)	50.8 (11.3)		0.3 (0.1,0.4)**			

Note. Negative effect sizes indicate a reduction in symptoms. * p<.05; **p<.01; ***p<.001; ***Abbreviations***. SD = Standard deviation; CI = Confidence interval; PSS=Perceived Stress Scale; eGPC = enhanced group prenatal care; eIPC = enhanced individual prenatal care. [1]Estimated standardized change in mean values over time within a group, adjusted for baseline value of outcome, self-reported history of a mental health condition, calendar time at enrollment, and language of the questionnaire. [2]Standardized difference between groups in estimated change over time in mean values, adjusted for baseline value of outcome, self-reported history of a mental health condition, calendar time at enrollment, and language of the questionnaire

© 2026 Felder JN et al. *JAMA Network Open*.
