# Supplementary material for: Enhanced Prenatal Care Models and Postpartum Depression: The EMBRACE Randomized Clinical Trial
Source: JAMA Netw Open. 2026 Feb 16;9(2):e2559883. doi: 10.1001/jamanetworkopen.2025.59883 (PMC12910397; doi:10.1001/jamanetworkopen.2025.59883)
Supplement: Supplement 3. — Data Sharing Statement [file jamanetwopen-e2559883-s003.pdf]

Data Sharing Statement

Felder. Enhanced Prenatal Care Models and Postpartum Depression. *JAMA Netw Open*.
Published February 16, 2026. doi:10.1001/jamanetworkopen.2025.59883

Data
Data available: Yes
Data types: Deidentified participant data, Data dictionary
How to access data: miriam.kuppermann@ucsf.edu
When available: With publication

Supporting Documents
Document types: Statistical/analytic code
How to access documents: miriam.kuppermann@ucsf.edu
When available: With publication

Additional Information
Who can access the data: researchers whose proposed use of the data has been approved
Types of analyses: for research that has been approved
Mechanisms of data availability: With a signed data use agreement
